# Include or not to include conference abstracts in systematic reviews? Lessons learned from a large Cochrane network meta-analysis including 585 trials

**DOI:** 10.1186/s13643-022-02048-6

**Published:** 2022-08-26

**Authors:** Samantha Hackenbroich, Peter Kranke, Patrick Meybohm, Stephanie Weibel

**Affiliations:** Department of Anaesthesiology, Intensive Care, Emergency and Pain Medicine, University Hospital Wuerzburg, 97080 Wuerzburg, Germany

**Keywords:** Randomized controlled trial, Conference abstract, Systematic review, Reporting quality, CONSORT, Postoperative nausea and vomiting

## Abstract

**Background:**

Systematic reviews attempt to gather all available evidence. Controversy exists regarding effort and benefit of including study results presented at conferences only. We recently published a Cochrane network meta-analysis (NMA) including 585 randomized controlled trials comparing drugs for prevention of postoperative nausea and vomiting (PONV). Studies published as conference abstracts only were excluded. This study aimed to include all eligible studies published as abstracts only, assessing their added value regarding reporting quality and effect on the review’s interpretation.

**Methods:**

Conference abstracts were searched in the review’s excluded studies and conference proceedings of anaesthesiologic societies. We assessed their reporting quality regarding review’s eligibility criteria, Cochrane ‘risk of bias’ assessment tool 1.0, and adherence to CONSORT (Consolidated Standards of Reporting Trials) for abstracts. Abstracts were included in sensitivity NMA, and impact on the NMA structure was investigated.

**Results:**

We identified 90 abstracts. A total of 14% (13/90) were eligible. A total of 86% (77/90) are awaiting classification due to insufficient reporting of review’s eligibility criteria. In abstracts awaiting classification, sufficient information was missing on standardization of anaesthesia in 71% (55/77), age of participants in 56% (43/77), and outcome details in 46% (36/77). A total of 73% (66/90) of abstracts lacked sufficient information on 15/25 data extraction items. Reported study characteristics of abstracts were comparable to included studies of the review. A total of 62% (56/90) of abstract trials were assessed as overall high risk of bias due to poor reporting. Median adherence to CONSORT for abstracts was 24% (*IQR*, 18 to 29%). Six of the 13 eligible abstracts reported relevant outcome data in sufficient detail for NMA on seven outcomes of the Cochrane review. Inclusion of abstracts did not substantially change the network structure, network effect estimates, ranking of treatments, or the conclusion. Certainty of evidence for headache on palonosetron use was upgraded from very low to low.

**Conclusions:**

Most conference abstracts on PONV were insufficiently reported regarding review’s narrow inclusion criteria and could not be included in NMA. The resource-intensive search and evaluation of abstracts did not substantially extent the full-text evidence base of the review, given the few adequately reported abstracts. Conferences should oblige authors to adhere to CONSORT for abstracts.

**Supplementary Information:**

The online version contains supplementary material available at 10.1186/s13643-022-02048-6.

## Background

Systematic reviews attempt to gather all available evidence, including unpublished studies on a specific research question. Controversy exists regarding the added value of including study results presented at conferences only [1]. While Cochrane highly desires searching relevant grey literature sources when generating a review [2], the US Agency for Healthcare Research and Quality only recommends that these searches be considered [3]. However, searching for and including conference abstracts are resource intensive [1]. In contrast to traditional systematic reviews with their comprehensive data pool, rapid reviews apply methodological short cuts, like limiting grey literature searches, to generate the review in a shorter time [4, 5].

Including conference abstracts in systematic reviews can increase its comprehensiveness and precision and can decrease the potential impact of publication bias [1, 6]. Other meta-epidemiologic studies have shown only minor differences in results between meta-analyses with and without conference abstracts [7, 8]. A Cochrane review has shown that one-third of the randomized controlled trials (RCTs) presented at conferences are subsequently not published in full [6]. They found evidence for publication bias as probability for full publication depended on the direction of the results [6].

Information in abstracts is often inadequately reported, and their dependability is questionable [1, 9–14]. When abstracts are the only record of the study results, providing all necessary information in abstracts to appraise the study and assess the eligibility for a systematic review is fundamental to increase the value for evidence synthesis. A crucial step towards higher reporting quality was the introduction of reporting guidelines like the CONSORT (Consolidated Standards of Reporting Trials) statement including a special version for journal and conference abstracts, published in 2008 [15].

The recent Cochrane review with network meta-analysis (NMA) from Weibel and colleagues compared the efficacy and safety of antiemetic drugs for the prevention of postoperative nausea and vomiting (PONV) in adults after general anaesthesia [16]. To enhance feasibility of the workload with an estimated number of more than 1000 trials for full-text screening, all trials published as conference abstracts only were excluded in the review protocol [16]. The current study addressed retrospectively whether inclusion of all eligible studies published as abstracts only is able to change the review’s interpretation. To this end, we searched for additional conference abstracts that were eligible for the Cochrane review and assessed abstracts’ reporting quality, risk of bias, and the impact on the conclusion of the review.

## Methods

This study is a case study on the impact of randomized controlled studies published as conference abstracts only and which met otherwise the narrow eligibility criteria of the large Cochrane review published by Weibel et al. [16, 17]. This study has not been registered. A protocol for the retrospective case study has not been prepared. A completed PRISMA 2020 checklist is available in Additional file 1.

### Eligibility criteria

For this study, RCTs reported as conference abstracts without a subsequent full-text publication were eligible only. No language restrictions were applied. The prespecified narrow eligibility criteria of the Cochrane review were applied for this study [16]. In brief, studies were required to investigate adult participants (i.e. the majority of participants over 18 years of age) undergoing any type of surgery with general anaesthesia and compare single or multiple pharmacological intervention(s) with antiemetic action belonging to one of the six drug classes (5-HT_3_ receptor antagonists, D_2_ receptor antagonists, NK_1_ receptor antagonists, corticosteroids, antihistamines (histamine 1 receptor antagonists), and anticholinergics) versus each other, versus no treatment, or versus placebo. All drugs had to be administered before or during anaesthesia to prevent PONV. Participants in all treatment arms within a study must have been subject to the same anaesthesia regimen. Primary outcomes of the review were vomiting within 24 h postoperatively, serious adverse events (SAEs), and any adverse event (AE) both within 7 days postoperatively. Secondary outcomes were drug class-specific side effects (e.g. headache, constipation, extrapyramidal symptoms, sedation, arrhythmia, QT prolongation, wound infection, and visual disturbances), early and late vomiting, nausea, and complete response (CR, no nausea and no vomiting, and no rescue antiemetic treatment for the first 24 h).

### Information sources and search strategy

We searched the list of excluded studies from the Cochrane review for eligible abstracts. The review’s search included the Cochrane Central Register of Controlled Trials (CENTRAL), MEDLINE, Embase, CINAHL, and study registers (ClinicalTrials.gov, WHO ICTRP) and reference lists of relevant systematic reviews. Details of the search strategy are provided in the full Cochrane review [16].

Additionally, we searched the online available conference proceedings of the American Society of Anesthesiologists (ASA, 2000–2017), European Society of Anaesthesiology (ESA, 2004–2017), and International Anesthesia Research Society (IARS, 2012–2017) for eligible abstracts. Search keywords were ‘PONV’, ‘vomit’, ‘nausea’, ‘emesis’, and ‘emetic’.

Conference abstracts published up to November 2017, the primary search date of the Cochrane review, were eligible. If a full-text publication was found, we excluded the abstract. Duplicates were removed.

### Study selection and data extraction

Abstracts were assessed for eligibility, and data were extracted by a single reviewer using Covidence [18]. Critical issues regarding eligibility or study details were discussed with a second reviewer.

If information on eligibility (PICO) criteria, including details on participants (P), intervention (I), comparator (C), and outcome (O), was missing or insufficiently reported, abstracts were categorized as awaiting classification. The number of missing or insufficient information regarding any eligibility criteria per abstract awaiting classification was determined. We compared the reasons for assessment as ‘awaiting classification’ before and after 2008, the year of the introduction of CONSORT for abstracts.

Data extraction followed a predefined and piloted data extraction form. In brief, we extracted the literature source, year of publication, publication language, the location of the study conduct/origin of authors (Africa, Asia, Australia, Europe, North America, South America), and setting (multicentre, single centre). Source of funding (sponsored by industry, nonindustry resources, not reported) and trial registration number were identified. Type of surgery, intervention details (inactive control, only active arms, single interventions, combination interventions), and the study’s outcomes with details were further extracted. We compared the characteristics of the eligible abstracts with those awaiting classification and with included studies of the Cochrane review.

### Risk of bias

We assessed the risk of bias of eligible abstracts, and of those awaiting classification using the Cochrane ‘risk of bias’ assessment tool 1.0, and summarized the overall risk of bias for each study by reference to the judgements of the domains ‘sequence generation’, ‘blinding of participant, personnel, and outcome assessors’, and ‘incomplete outcome data’. We compared the risk of bias assessments of the abstracts with those of included studies in the Cochrane review. Methodological details of risk of bias assessment were described in the Cochrane review [16].

### Assessment of reporting quality in abstracts

We assessed the reporting of data relevant for specific data extraction according to the Cochrane review extraction sheet with 25 relevant items in both eligible abstracts and abstracts awaiting classification. The analysed categories were study identification, study design and details regarding study populations, intervention, and outcomes.

We further assessed the reporting of relevant information necessary for risk of bias assessment. Information per item was judged as ‘sufficient’, ‘insufficient’, or ‘missing’.

Furthermore, we assessed the adherence to CONSORT for abstracts checklist items as ‘sufficient’, ‘insufficient’, or ‘missing’. We compared the reporting of data, risk of bias relevant information, and CONSORT for abstracts items in abstracts before and after 2008, the year of the introduction of CONSORT for abstracts.

### Contribution of abstracts to network meta-analyses

Eligible abstracts with relevant outcome data that were reported in sufficient detail were assessed for their impact on the primary NMA. First, we investigated whether inclusion of abstracts changed the network information and structure, e.g. addition of new studies, new treatments, and new direct or indirect comparisons. Second, in sensitivity analyses, eligible abstracts were added to the primary NMAs published in the Cochrane review [16, 17]. Details for NMA were described elsewhere [16, 17]. We compared NMA effect estimates and ranking order of treatments of the primary NMA and the sensitivity analysis. We classified a relevant change in NMA effect estimates if the estimated effects differed with respect to the range of equivalence (i.e. was located outside the predefined range of equivalence), which was set as a risk ratio (RR) of 0.8 to 1.25 for PONV-related outcomes and a RR of 0.9 to 1.11 for all safety outcomes.

## Results

**Fig. 1:**
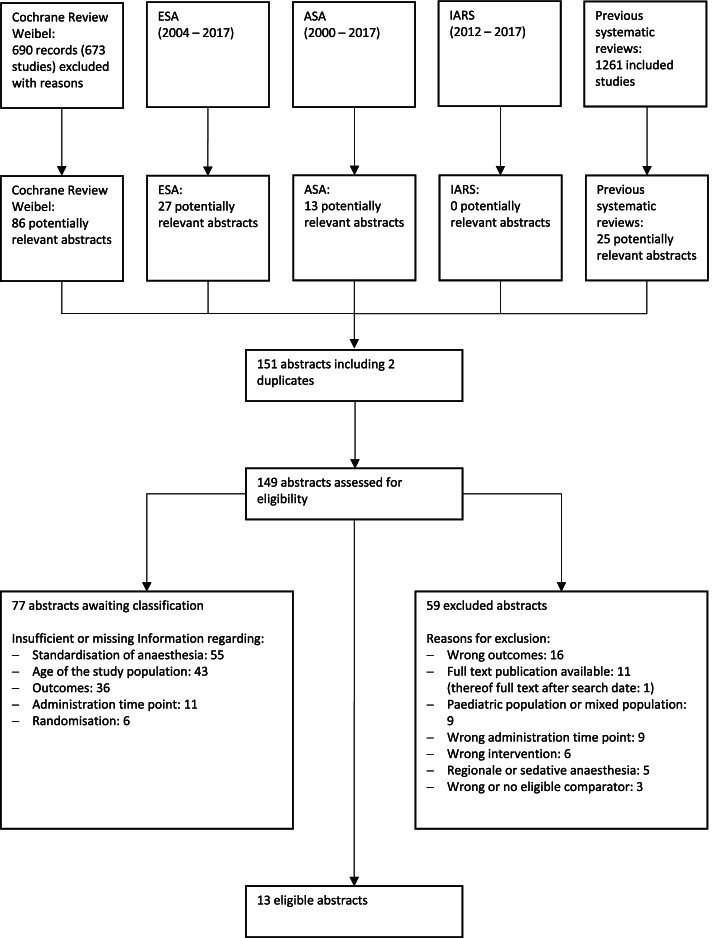
Study flow diagram. ASA (American Society of Anesthesiologists), ESA (European Society of Anaesthesiology), and the IARS (International Anesthesia Research Society)

We identified 151 potentially relevant abstracts by title screening from different sources (Fig. 1). After duplicates had been removed, 149 abstracts were assessed in detail for eligibility, and 59 abstracts were excluded with reasons (Fig. 1). Of the remaining 90 studies, 13 abstracts (14%) were eligible for inclusion, and 77 abstracts (86%) are awaiting classification due to lack of information regarding relevant eligibility (PICO) criteria. References to eligible abstracts and abstracts awaiting classification are available in Additional file 2. About half of all eligible abstracts (46%) and the abstracts awaiting classification (45%) were retrieved from the list of excluded studies in the Cochrane review (Additional file 3).

In 86% of the abstracts (77/90), full eligibility could not be assessed due to insufficient or missing information on PICO criteria. Among the most frequent reasons leading to the assessment as ‘awaiting classification’ was a lack of information on the standardization of the anaesthesia regimen in the study arms (71%), age of participants (56%), and outcome details (47%) (Table 1). In 65% of all abstracts, awaiting classification (50/77) had more than one missing information regarding any eligibility criteria (Table 1). Comparing abstracts published before and after publication of the CONSORT statement for abstracts in 2008 revealed no difference in the availability of information on relevant eligibility criteria (Table 1).

**Table 1:** Reasons for awaiting classification (abstracts)

Inclusion (PICO) criteria	Insufficient/missing information		
	All abstracts awaiting classification (*n* = 77)	Abstracts before 2008 (*n* = 43)	Abstracts since 2008 (*n* = 34)
Standardization of anaesthesia	55 (71%)	27 (63%)	28 (82%)
Age of participants	43 (56%)	26 (60%)	17 (50%)
Outcome details	36 (47%)	20 (47%)	16 (47%)
Time point of administration	11 (14%)	2 (5%)	9 (26%)
Study design (e.g. randomization)	6 (8%)	5 (12%)	1 (3%)
Number of insufficient/missing inclusion criteria per abstract	-	-	-
1	27 (35%)	15 (35%)	12 (35%)
2	27 (35%)	17 (40%)	10 (29%)
3	18 (23%)	10 (23%)	8 (24%)
4	5 (6%)	1 (2%)	4 (12%)

We compared the characteristics of studies published as eligible abstracts and abstracts awaiting classification with included studies of the Cochrane review (Additional file 3). Overall, the study characteristics were comparable with some minor differences pointed out in the following. Most studies were published in English and were primarily conducted in Asia, Europe, or North America. However, the majority of studies included in the Cochrane review were from Asia (51%), while eligible abstracts were mostly from North America (46%). Most studies investigated single drugs only, mostly ondansetron. The majority of the 13 eligible abstracts compared active interventions only (62%), whereas the majority of the 585 included studies in the Cochrane review had at least one inactive control arm (66%). Most participants were women. However, the proportion of women in the Cochrane review was higher (88%) than in abstracts (49% and 53%). The application of perioperative opioids was less frequently reported in abstracts than in studies included in the review. In the majority of abstracts, the type of general anaesthesia was not specified, in contrast to 88% using volatile anaesthetics in studies included in the review. Overall, most participants underwent gynaecological surgery. However, neurological procedures were more frequent in eligible abstracts (23% vs. 15% gynaecological procedures).

Overall, reporting of data relevant for data extraction according to the Cochrane review´s extraction sheet (25 items) was poor in eligible abstracts and abstracts awaiting classification. A total of 73% of abstracts (66/90) had missing or insufficient information on 15 out of 25 data extraction items. Eight of 25 categories were not addressed in more than 80% of all abstracts (Additional file 4). There is no overall improvement in reporting of data in abstracts published before and after 2008 (Additional file 4).

**Fig. 2:**
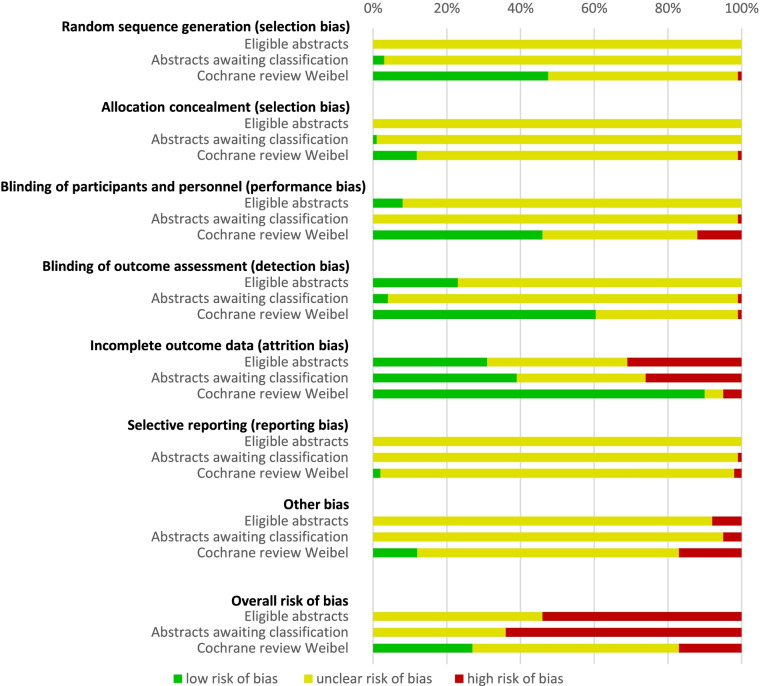
Comparison of Cochrane’s risk of bias assessment for abstracts and studies of the Cochrane review

The risk of bias assessment of studies differed between studies published as abstracts, eligible or awaiting classification, and full-text publications, included in the Cochrane review (Fig. 2). None of the abstracts was assessed as overall low risk of bias compared to 27% of studies included in the Cochrane review. A total of 62% of all abstracts (56/90) were assessed as overall high risk of bias, including 7/13 of the eligible abstracts and 49/77 of the abstracts awaiting classification.

Reporting of relevant information for risk of bias assessment on individual domains was inadequate. A total of 96% of all abstracts (86/90) had insufficient or missing information in at least six out of seven risk of bias domains resulting in an ‘unclear risk of bias’ assessment (Additional file 5). Most details were reported for the domain ‘incomplete outcome data’ with sufficient information in 38% of the abstracts. In general, there was no difference in the reporting of relevant information for risk of bias assessment in abstracts published before and after 2008 (Additional file 5).

Adherence to the CONSORT for abstracts reporting items was also suboptimal, with a median adherence per abstracts of 4 out of 17 items (24%) (interquartile range: 3 to 5 (1 to 29%)). None of the abstracts provided sufficient contact details, methods of sequence generation and allocation concealment, and adequate outcome results (Additional file 6). We also found very low adherence to describing blinding (2%) and trial registration (1%). There is no overall improvement in reporting according to CONSORT for abstract items in abstracts that were published before and after 2008 (Additional file 6).

Six of the 13 eligible abstracts reported relevant outcome data in sufficient detail for network meta-analyses on seven outcomes of the Cochrane review (Table 2). In the remaining seven studies, relevant information for data analysis was missing. Three studies could be included in the NMA of nausea within 24 h; two studies each were included in the NMA of vomiting within 24 h, early vomiting, complete response, and headache; and one study was included in the NMA of QT prolongation and vomiting late (Table 3, Additional file 7). The network structure of these outcomes was hardly affected. Inclusion of abstracts added one new treatment dolasetron-scopolamine in comparison with dolasetron to the outcomes early vomiting and late vomiting, and one new direct comparison droperidol-metoclopramide versus ondansetron was added to the outcome early vomiting (Table 3, Additional file 7). The number of additional comparisons per NMA due to inclusion of abstracts ranged between one and 11 (Table 3, Additional file 7). Effect estimates from single abstract studies in comparison with NMA effect estimates are shown in the Additional file 7. Inclusion of abstracts in sensitivity NMA analyses did not change the primary network effect estimates nor the ranking of treatments for any of the seven outcomes in a relevant manner (Table 3, forest plots are shown in Additional file 8). For most effect estimates, the clinical interpretation of effects did not change, with exception of palonosetron for vomiting within 24 h and headache. In case of vomiting, the clinical interpretation of the effect of palonosetron changed from uncertain (*RR* 0.62, 95% *CI* 0.48 to 0.80) to important benefit (*RR* 0.63, 95% *CI* 0.50 to 0.79), as the 95% CI did no longer include the range of equivalence (95% *CI* 0.80 to 1.25) (Table 3). However, certainty of evidence remained low. For headache, the certainty that palonosetron increases the risk for headache was upgraded from very low to low.

**Table 2:** Outcomes for analyses in eligible abstracts (*n* = 13)

Eligible abstracts	Primary outcomes		Secondary outcomes						
	Vomiting 0–24 h	Any AE	Headache	Extrapyramidal symptoms	QT prolongation	Early vomiting	Late vomiting	Nausea	Complete response
Cardone 2007	-	-	-	-	-	-	-	?	-
George 2016	-	-	-	-	-	-	-	x	-
Ghosh 2009	-	-	-	-	-	?	?	-	-
Huston 1996	-	?	-	-	-	?	-	-	-
Ilbeigi 1999	x	-	x	-	-	-	-	x	-
Kathirvel 1998	?	-	-	-	-	-	-	?	-
Meyer 2004	-	-	-	-	-	x	x	-	-
Naco 2012	-	-	-	-	-	?	?	-	-
Samra 2003	-	-	-	-	-	-	-	x	x
Sansone 2011	-	-	-	?	-	-	-	?	-
Sousa 2014	?	-	-	-	-	-	-	?	-
Sun 1995	-	-	-	-	-	x	-	-	-
White 2005	x	-	x	-	x	-	-	-	x

‘x’ sufficiently reported, ‘-’ not reported, ‘?’ missing information. Outcomes without new studies (abstracts): SAE, constipation, sedation, arrhythmia, wound infection, visual disturbances

**Table 3:** Sensitivity NMA analyses of NMA results primary and secondary outcomes including eligible abstracts

Outcome	Number of studies (new studies)§	Number of treatments (new treatments)	Number of pairwise comparisons (new comparisons)	Ranking^a^ treatment: new rank (+/−number of rank)	RR (95% CI)^b^	
					Without abstracts	With abstracts
Vomiting 24 h	284 (+2) Ref. [5, 13]	65 (+0)	576 (+4)	NA	Palo RR 0.62 (0.48 to **0.80**)	Palo RR 0.63 (0.50 to **0.79**)
Headache	210 (+2) Ref. [5, 13]	53 (+0)	430 (+4)	Dexa-meto-onda: 16 (−8) Meto-onda: 30 (−7)	Meto-onda *RR* **0.77** (0.18 to 3.19) Palo *RR* 1.15 (**0.89** to 1.49)	Meto-onda *RR* **0.94** (0.29 to 3.03) Palo *RR* 1.16 (**0.91** to 1.49)
QT prolongation	19 (+1) Ref. [13]	13 (+0)	38 (+1)	NA	NA	NA
Vomiting ‘early’	265 (+2) Ref. [7, 12]	70 (+1)	575 (+11)	Perp: 31 (−12) Drop-meto: 42 (+10)	NA	NA
Vomiting ‘late’	207 (+1) Ref. [7]	52 (+1)	439 (+1)	NA	NA	NA
Nausea	327 (+3) Ref. [2, 5, 9]	65 (+0)	680 (+10)	Dime: 38 (+6)	NA	NA
Complete response	138 (+2) Ref. [9, 13]	38 (+0)	285 (+7)	NA	NA	NA

§References from Additional file 2, eligible abstracts. ^a^Ranking: we named substantial changes > 5 only. ^b^Changes in the interpretation of effects according to the range of equivalence. For vomiting, nausea, and complete response, the range of equivalence is *RR* 0.8 to 1.25; for headache and QT prolongation, it is *RR* 0.9 to 1.11. *CI* confidence interval, *NA* not applicable, *Ref*. reference, *RR* risk ratio, *dexa* dexamethasone, *dime* dimenhydrinate, *drop* droperidol, *meto* metoclopramide, *onda* ondansetron, *palo* palonosetron, *perp*, perphenazine

## Discussion

Reporting in abstract publications in clinical PONV research is poor. Thus, we could not include the majority of abstracts into the recent Cochrane review on PONV with narrow eligibility criteria. In contrast, only 12% (206/1762) of full-text records assessed for eligibility in the Cochrane review were awaiting classification due to missing information in the study reports or study protocols [16]. Previous systematic reviews on PONV had broader inclusion criteria [19–21]. Therefore, more conference abstracts might be eligible, despite their deficiencies in reporting.

Overall, study characteristics of abstracts were mostly comparable to the included studies in the review. Interestingly, 62% of eligible abstracts compared active interventions only, whereas most of the studies (66%) in the review had an inactive control arm. This might indicate a publication bias since studies comparing active drugs might be prone to nonsignificant results. However, publication bias played a minor role in this field of clinical research as we suspected publication bias for only seven out of 64 direct comparisons with more than 10 RCTs in the NMA [16]. Moreover, considering both eligible abstracts and those awaiting classification, 49% compared active interventions only, while 51% had an inactive control arm. Therefore, the difference between eligible abstracts and studies in the review is probably only a random effect due to the limited data pool of 13 eligible abstracts. Abstracts were more frequently assessed as overall high risk of bias, due to poor reporting [16].

Complete, transparent, and accurate reporting in conference abstracts is fundamental as the conference abstract may remain the only available record of that trial [6]. However, reporting was inadequate across all areas analysed: eligibility criteria, study details for specific data extraction, risk of bias assessment, and CONSORT for abstracts checklist. Previous studies on reporting of abstracts in anaesthesia clinical trials mainly focused on the adherence to CONSORT of journal abstracts [22–24]. However, in the field of pain research, the reporting of conference abstracts has been assessed, from 2008 to 2014 [11]. With 26%, median adherence to CONSORT for abstracts was similar to this study (24%) [11]. In both studies, no abstract adequately reported contact details, and only one each mentioned trial registration [11]. The lack of e-mail address and study registration number is particularly concerning in conference abstracts, as they are important ways to obtain further study information [1, 25]. With 2% and 6% respectively, Saric and colleagues found very low adherence regarding randomization and recruitment status, comparable to ours (0% and 4%, respectively) [11]. Similar to our study, poor methodological reporting was found in 1992 and 2002 oncologic conference abstracts regarding method of allocation concealment (1%) and of blinding (12%) [14]. Overall, reporting needs to improve as fellow researchers have found [10, 14].

It is difficult to report in abstracts comprehensively when the total number of words or headings within an abstract is strictly limited. However, 250 to 300 words are suffice to address the CONSORT for abstracts checklist [15]. The 2020 instructions for authors of ASA, ESA, and IARS conferences permitted at least 2500 characters including spaces [26–28]. Previous of these gatherings, and conferences not explicitly referred to here, may have imposed different restrictions.

Overall, in our study, reporting did not improve after the publication of the CONSORT statement for abstracts in 2008. Our analysed “post-CONSORT” time period beginning in 2008 might be too early to capture the impact of CONSORT for abstracts. At that time, CONSORT might not have gained sufficient awareness yet. In addition, lack of improvement of reporting may be due to the missing requirement at conferences, for authors to adhere to CONSORT for abstracts. Still, 2020 instructions for authors of the ASA, ESA, and IARS conference proceedings do not mention any reporting guideline, in contrast to the journals *Anesthesiology*, *European Journal of Anaesthesiology*, and *Anesthesia & Analgesia* [26–31]. Only two of 819 instructions for abstracts on conferences had reporting instructions for RCT or systematic review authors [32]. Studies on implementation of CONSORT in journals have shown a potential positive influence on reporting [33, 34]. Thus, authors should be required to adhere to CONSORT for abstracts when submitting a conference abstract [11].

Inclusion of eligible abstracts in the NMA did not substantially change the network structure, network effect estimates, ranking of treatments, or the conclusion. The interpretation of the estimated benefit of palonosetron for vomiting within 24 h changed from ‘uncertain’ to ‘important’, but certainty of evidence remained low. Certainty of evidence for palonosetron boosting the risk of headache increased from ‘very low’ to ‘low’. All changes are of minor clinical relevance. As we could only include study results of six abstracts in the sensitivity NMA due to poor reporting in abstracts of inclusion criteria and outcome details, major differences to the original NMA with 585 studies could not have been expected. However, based on the amount of full-text studies, we did originally expect to find a greater number of eligible abstracts with a potentially greater impact on the NMA. Our case study supports the suggestion by Scherer et al. to consider availability of evidence informing a review, when deciding whether or not to search for conference abstracts [1]. As the full-text evidence on prevention of PONV research is plentiful [16], the added value of including conference abstracts appears to be limited. This finding may also be relevant to the generation of rapid reviews. Our study may indicate that limiting grey literature searches may be a reasonable methodological short cut, to generate a review in a shorter time. It is also supported by the guidance on rapid reviews from Cochrane and the WHO and Alliance for Health Policy and Systems Research [4, 5]. Our findings are consistent with a meta-epidemiological study identifying abbreviated literature searches as viable options for rapid evidence syntheses, when less certainty and a small risk for opposite conclusions can be accepted [35]. A study on the contribution of non-English reports and unpublished studies also showed only rarely an impact on the conclusion in child-relevant reviews [7].

This study has several limitations.

First, the study was performed in a single reviewer mode, including the abstract search in conference proceedings and assessment of eligibility and reporting. Despite all care and attention, including discussion with a second reviewer, studies might have been missed or have been inconsistently assessed. Assessment of reporting is always subjective, and identified inadequacies in studies’ reporting do not necessarily correspond to poor methodological study quality. Second, our study may underrepresent conference proceedings in a language other than English. We searched three English-language conference proceedings. However, most of the abstracts were retrieved from the list of excluded studies of the review, which did not apply any language restrictions. Third, the small number of sufficiently reported abstracts is the main limitation that prevents an impact on the conclusion of the NMA. We did not contact any author for missing abstract information, as reporting quality was a matter of analysis. Thus, more abstracts may be fully eligible, with a potentially greater impact on the NMA.

## Conclusions

In summary, most conference abstracts on PONV were insufficiently reported regarding review’s narrow inclusion criteria and therefore could not be included in the NMA. The resource-intensive search and evaluation of abstracts did not substantially extend the already solid full-text evidence base of the review, given the small number of adequately reported abstracts. If in conference proceedings authors are obliged to adhere to CONSORT for abstracts, using clinical trial abstracts in systematic reviews may yield a more relevant evidence gain in the future.

## Supplementary Information

**Additional file 1.** Completed PRISMA 2020 checklist.

**Additional file 2.** References to eligible abstracts and abstracts awaiting classification.

**Additional file 3.** Study characteristics of eligible abstracts, abstracts awaiting classification and included studies in the review; data sheet with the study characteristics of eligible abstracts, abstracts awaiting classification and included studies in the review.

**Additional file 4.** Reporting of data for data extraction according to the Cochrane review’s extraction sheet (25 items); data sheet with the assessment of reporting quality of all abstracts regarding data extraction items.

**Additional file 5.** Reporting of information for risk of bias assessment in abstracts; data sheet with the assessment of reporting quality of all abstracts regarding risk of bias relevant information.

**Additional file 6.** Adherence to CONSORT for abstracts reporting items; data sheet with the assessment of reporting quality of all abstracts regarding the CONSORT for abstracts checklist.

**Additional file 7.** Effect estimates of antiemetic treatments in the abstracts and the NMA; data sheet comparing effect estimates of antiemetic treatments in the abstracts vs. review (direct evidence/NMA).

**Additional file 8.** Forest plots NMA; Sensitivity analyses including abstracts; graphical presentation of the NMA effect estimates of treatments per outcome with and without abstracts.

## Data Availability

The datasets used and/or analysed during the current study are available from the corresponding author on reasonable request.

## Abbreviations

AE: Adverse event
ASA: American Society of Anesthesiologists
CENTRAL: Cochrane Central Register of Controlled Trials
CONSORT: Consolidated Standards of Reporting Trials
CI: Confidence interval
CINAHL: Cumulative index to nursing and allied health literature
CR: Complete response
ESA: European Society of Anaesthesiology
NMA: Network meta-analysis
PICO: Participants, intervention, comparator, and outcome
PONV: Postoperative nausea and vomiting
RCT: Randomized controlled trial
RR: Risk ratio
sAE: Serious adverse events
WHO: World Health Organisation
