# Supplementary material for: Include or not to include conference abstracts in systematic reviews? Lessons learned from a large Cochrane network meta-analysis including 585 trials
Source: Syst Rev. 2022 Aug 26;11:178. doi: 10.1186/s13643-022-02048-6 (PMC9413929; doi:10.1186/s13643-022-02048-6)
Supplement: Supplementary file 2 — Additional file 2. References to eligible abstracts and abstracts awaiting classification. [file 13643_2022_2048_MOESM2_ESM.docx]

**Supplementary File 2**

References to eligible abstracts and abstracts awaiting classification

Eligible abstracts

1. Cardone A, Aceto, P., Congedo, E., Chinè, E., De Cosmo, G. Effects of ondansetron and acupuncture on postoperative nausea and vomiting. Eur J Anaesthesiol. 2007;24(Suppl 39):121-2.

2. George J, Olton, S., Hariharan, S., Chen, D. The effectiveness of P6 acupressure bands in treating post-operative nausea and vomiting. Anesth Analg. 2016;123(3 Suppl 2):469.

3. Ghosh JS. Comparison of granisetron and ondansetron for prevention of PONV in patients undergoing elective craniotomy - a randomized, double-blind study. J Neurosurg Anesthesiol. 2009;21(4):388.

4. Huston CL, Sheridan, C.A., Ungard, S.D., Selley, K., Hagan, A., Nagelhout, J., et al. Comparison of oral granisetron, intravenous granisetron, and droperidol in the prevention of nausea and vomiting after outpatient laparoscopic procedures. AANA J. 1996;64(5):437-8.

5. Ilbeigi MS, Ponnudurani, R., Liu, P. A comparison of the efficacy of ondansetron or metoclopramide or ondansetron and metoclopramide in the prevention of postoperative nausea and vomiting. Anesth Analg. 1999;88:s348.

6. Kathirvel S, Balachundhar, S., Bhatia, A., Dash, H.H., Chaturvedi, A. Postoperative nausea and vomiting after elective craniotomy. Anesthesiology. 1998;89:A376. [3220491; SI:000075810900376 ER].

7. Meyer TA, Roberson, C.R., McAllister, R.K., McKinney, R., Rajab, M.H. Dolasetron and transdermal scopolamine vs. dolasetron for prevention of postoperative nausea and vomiting in outpatients undergoing laparoscopic surgery: a randomized, double-blind comparison. Anesthesiology. 2004;101:A75.

8. Naco M, Mandi, A., Gani, H., Llukacaj, A., Kodra, N., Rakipi, B. The efficacy and safety of ondansetron, dexamethasone and droperidol in preventing postoperative nausea and vomiting (PONV) after thyroidectomy. Eur J Anaesthesiol. 2012;29:13.

9. Samra SK, Williams, B., Ciarralo, C., Turner, C., Smythe, P. Lack of effect of droperidol, ondansetron and dolasetron administration on PONV in a placebo controlled trial. Anesthesiology. 2003;99:A305.

10. Sansone P, Stumbo, R., D'Arienzo, S., Coletta, F., Zagaria, G., Pace, M.C., et al. Droperidol vs metoclopramide for prophylaxis of postoperative nausea and vomiting after breast cancer surgery. Eur J Anaesthesiol. 2011;28:141.

11. Sousa AM, Chaves De, M.L., Magalhaes, N.G.G., Fagundes, F.G., Adel, A.H. Addiction of neurokinin 1 receptor antagonist aprepitant to double antiemetic prophylaxis improves control of postoperative nausea and vomiting in oncologic patients undergoing laparoscopic procedures: a double-blinded randomized controlled trial. Eur J Anaesthesiol. 2014;31:155.

12. Sun R, Klein, K., White, P.F. Use of ondansetron, metoclopramide and droperidol for preventing postoperative nausea and vomiting after ent surgery. Anesthesiology. 1995;83:A20.

13. White PF, Scuderi, P. Prevention of postoperative nausea and vomiting (PONV): A dose-ranging study involving palonosetron, a potent 5-HT3 receptor antagonist. Anesthesiology. 2005;103:A703.

Abstracts awaiting classification

1. Abbasi S, Talakoub, R., Shokrieh, A. Effect of betamethasone on postoperative analgesic and antiemetic requirement. Pain Pract. 2009;9:139.

2. Aguila C, Leyton, P., Volpato, G., Lagos, P., Cumsille, M. Is it necessary to use ondansetron during a total intravenous anesthesia with propofol to reduce the incidence of postoperative nausea and vomiting (PONV)? Eur J Anaesthesiol. 2004;21(Suppl 32):4.

3. Arslan G, Gureler, C., Ozden, A., Karamanlioglu, B., Pamukcu, Z. Role of tropisetron, granisetron and dexamethasone in prophylaxis of nausea and vomiting seen after gynaecological operations. Eur J Anaesthesiol. 2000;17(Suppl 19):30.

4. Bhutia J, Stolte, C., Dedecker, J., Lauwers, M., Poelaert, J. Comparison of droperidol and alizapride in the prevention of nausea and vomitiing in orthopedic patients using patient-controlled analgesia (PCA) piritramide for postoperative pain. Pain Pract. 2009;9:140-1.

5. Brady M, Sollo, D. Metoclopramide does not decrease the incidence of nausea after outpatient orthopedic surgery with propofol anesthesia. Anesthesiology. 1994;81(3A):A20.

6. Byhahn C, Lotz, G., Mäser, D.A., Lischke, V., Westphal, K. Haloperidol or droperidol for antiemetic prophylaxis after thyroid surgery. A randomized, double-blinded clinical trial. Anesthesiology. 2003;99:A1289.

7. Cavanaugh JJ. Prophylaxis of postoperative nausea and vomiting: a clinical comparison of ondansetron and droperidol. AANA J. 1996;64(5):429-30.

8. Chamchad D, Medved, M., Gefen, J., Dunton, C., Horrow, J. Comparison of oral aprepitant and lower dose dexamethasone vs. aprepitant alone for preventing PONV. Proceedings of the 2010 Annual Meeting of the American Society of Anesthesiologists. 2010:A928.

9. Chang Y-J, Won-Suk, L., Kwang-Beom, L., Yun-Yl, C. Comparison of palonestron, granisetron, and ramosetron to prevent postoperative nausea and vomiting after laparoscopic gynecologic surgery: a prospective observational trial. Eur J Anaesthesiol. 2015;32(e-Suppl 53):37.

10. Charton A, Ruimy, A., Greib, N., Faitot, V., Meyer, N., Diemunsch, P. Incidence of akathisia after PONV prophylaxis with droperidol or ondansetron in outpatient surgery. A multicenter controlled randomized trial. Eur J Anaesthesiol. 2016;33(e-Suppl 54):312.

11. Chelly J, Melson, T., Pollock, J., Hantler, C. Oral RS-25259 prevents postoperative nausea and vomiting following laparoscopic surgery. Anesthesiology. 1996;85:A21.

12. Ciric A. Multimodal approach and TIVA anesthesia management decreases clinically important PONV in high risk patients undergoing laparoscopic cholecystectomy. Eur J Anaesthesiol. 2013;30(Suppl 51):8.

13. Cohen SE, Woods, W.A., Wyner, J. Antiemetic effect of metoclopramide and droperidol. Anesthesiology. 1981;55(3 Suppl):303.

14. Conesa A, Gracia, C., Pelavski, A., Suescun, M., Mateo, D., Manrique, S., Andreu, E., Lopez, R., Vilardell, E., Castellano, D., Viedma, S., Hervas, C. No differences between low dose of dexametasone and ondansetron in the prevention of postoperative nausea and vomiting in patients who undergo ambulatory laparoscopic gynaecological surgery. Eur J Anaesthesiol. 2005;22(Suppl 34):12-3.

15. Cucereanu-Badica I, Bradis, A.A., Grintescu, I. Dexamethasone – a good choice for PONV prevention in ENT surgery. Eur J Anaesthesiol. 2006;23(Suppl 37):15.

16. D’Angelo R, Goldstein, M.J., Gerancher, J.C., Owen, M.D. A comparison of bolus droperidol, metoclopramide; ondansetron and propofol on post operative nausea and vomiting following outpatient laparoscopic surgery. Anesthesiology. 1997;87:A50.

17. Das PC, Basu, S., Singh, R., Shukla, V.K. Role of dexamethasone in prevention of post operative nausea and vomiting after laparoscopic cholecystectomy. Indian J Gastroenterol. 2010;29(Suppl 1):A67-A8.

18. Diamond MJ, Bailey, D., McPhee, A. The gender-dependent pharmacodynamic difference in the antiemetic action of metoclopramide is a dose-related phenomenon. Can J Anaesth. 1988;35(3):65.

19. Dundee JW, Milligan, K.R., McKay, A.C. Influence of intraoperative acupuncture and droperidol on postoperative emesis. Br J Anaesth. 1988;61:117-8.

20. Eberhart L, Arndt, C., Lange, H., Wulf, H., Morin, A. Dexamethasone, rofecoxib or a combination of both for oral premedication before abdominal hysterectomy. A randomized placebo-controlled trial. Eur J Anaesthesiol. 2008;25(Suppl 44):195-6.

21. Fitzpatrick KTJ, Dundee, J.W., Ghaly, R.G. Comparison on the antiemetic action of manual and electro acupuncture with that of cyclizine and metoclopramide. Br J Anaesth. 1987;59:936-837.

22. Gackle H, Wagner, P., Eberhart, L.H., Georgieff, M., Lang, G.E., Lang, G.K. Prevention of postoperative nausea and vomiting after vitreo-retinal surgery under general anesthesia. Invest Ophthalmol Vis Sci. 1999;40:ARVO Abstract 4968.

23. Gallinger EY, Lichvantsev, V.V. Cost-effectiveness of prophylactic antiemetic therapy by laparoscopic surgery. Eur J Anaesthesiol. 2006;23(Suppl 37):11-2.

24. Gandhi KA, Kumar, A., Bala, I., Panda, N. Comparison of palonosetron with palonosetron and dexamethasone in the prevention of post operative nausea vomiting in patients undergoing middle ear surgery. Anesth Analg. 2016;123(3 Suppl 2):467.

25. Gani H, Ohri, I., Kerci, M., Cobani, E., Naco, M., Beqiri, V. Comparison of dexamethasone and metoclopramide effect, and the effect of them combined on PONV on urology. Eur J Anaesthesiol. 2011;28:140.

26. Garzón JC, Alonso, B., Barrio, E., Villoria, M.J., Vicente, J., Muriel, C. Prevention of postoperative nausea and vomiting in otological surgery. Ondansetron versus metoclopramide. Rev Esp Anestesiol Reanim. 1993;40(Suppl 1):14-5.

27. Gecaj-Gashi A, Hasani, A., Nikolova, Z., Agolli, L., Bukoshi, Z., Uka, S. Dexamethasone compared with metoclopramide in prevention of postoperative nausea and vomiting in orthognathic surgery. Eur J Anaesthesiol. 2012;29:13.

28. Glaser C, Lerche, A., Schreier, H., Bendtsen, A., Galgocy, M., Schindler, I. Prevention of postoperative nausea and vomiting with dixyrazine and metoclopramide: a randomized, double blind, comparative trial. Anaesthesiol Intensivmed Notfallmed Schmerzther. 1999;34(Suppl 2):117.

29. Gurha P, Kaur, R.D., Sanjay, R.R. Prophylactic intravenous palonosetron, granisetron and ondansetron in the prevention of post operative nausea and vomiting in laparoscopic surgeries. Br J Anaesth. 2012;108:376.

30. Hatridge JI, Meyer, T.A.., Roberson, C.R., Robert D., Bastron, R.D. Comparison of single vs. combination Antiemetic prophylaxis in outpatient laparoscopic procedures. Anesthesiology. 2003;99:A8.

31. Homaei MM, Khodadad, P.K. Comparison of intramuscular metoclopramide and subcutaneous hyoscine as prophylactic drugs for late post operative nausea and vomiting in viterectomy patients. Br J Anaesth. 2012;108:374-5.

32. Hunting A, Kjærsgaard, P., Hjelle, K., Skovlund, E., Aune, H. Nausea and vomiting after breast cancer surgery: prophylaxis with ondansetron vs metoclopramide. Acta Anaesthesiol Scand. 1997;41(Suppl 110):182.

33. Idali B, El Kettani, C., Haddad, F., Barrou, H. The use of betamethasone for preventing postoperative nausea and vomiting after thyroidectomy. Anesthesiology. 2002;96:A443.

34. Jemia FZ, Oureghi, S., Dridi, M., Lebbi, M.A., Bouguerra, C., Yousfi, M.A. A comparison of the combination of aprepitant and dexamethasone versus the combination of droperidol and dexamethasone for the prevention of postoperative nausea and vomiting. Eur J Hosp Pharm. 2017;24:A59.

35. Kim SI, Cho, A., Yoo, J.H., Kim, M.G., Kim, S.H. The additive interaction between ondansetron and dexamethasone for preventing postoperative nausea and vomiting in gynecologic surgery. Eur J Anaesthesiol. 2016;33(e-Suppl 54):114.

36. Kiourtzieva E, Papavramidis, T., Kesisoglou, I., Grossomanidis, V., Papavramidis, S. Low dose of droperidol in confronting postoperative nausea and vomiting in laparoscopic cholecystectomy: A clinical trial. Eur J Anaesthesiol. 2010;27(Suppl 47):10.

37. Kranke P, Apfel, C., Papenfuss, T., Rauch, S., Sefrin, P., Roewer, N. Effect of the timing of dimenhydrinate on postoperative nausea and vomiting after ENT-surgery. Acta Anaesthesiol Scand. 1997;41(Suppl 112):256.

38. Kranke P, Apfel, C., Papenfuss, T., Rauch, S., Sefrin, P., Roewer, N. Effect of the timing of droperidol on postoperative nausea and vomiting after ENT-surgery. Acta Anaesthesiologica. Acta Anaesthesiol Scand. 1997;41(Suppl 112):256.

39. Kumar N, du Plessis, H., Guraraj, P., Olson, J. Prospective randomized controlled trial of combination antiemetic prophylaxis in patients undergoing breast surgery. Eur J Anaesthesiol. 2005;22(34):6.

40. Kuypers M, Himpe, D., Eeckelaert, J.P., Theunissen, W., Vandermeersch, E. Droperidol reduces PONV to a more acceptable level after desflurane anaesthesia in laparoscopic surgery. Eur J Anaesthesiol. 2000;17(Suppl 19):12.

41. Kwak HJ, Choi, J.J., Jo, Y.Y., Kim, Y.B., Kim, J.Y. Antiemetic efficacy comparison of dexmedetomidine versus dexmedetomidine-dexamethasone in highly-susceptible patients: a prospective randomized placebo-controlled study. Eur J Anaesthesiol. 2017;34(e-Suppl 55):373.

42. Lee J, Kim, M., Kang, W. A randomized, double-blind, comparative study to evaluate the efficacy and safety of ramosetron injection of nausea and vomiting associated with patient-controlled analgesia in colorectal cancer patients undergoing laparoscopic colectomy. Dis Colon Rectum. 2015;58(5):e283-4.

43. Lee Y, Chen, A., Yang, Y.L., Ho, G.H., Liu, H.T., Lai, H.Y. The prophylactic antiemetic effects of ondansetron, propofol, and midazolam in female patients undergoing sevoflurane anaesthesia for ambulatory surgery. Eur J Anaesthesiol. 2005;22(Suppl 34):11-2.

44. Lee Y, Huang, P.K., Row, P.H. A comparative study of dexamethasone plus midazolam, ondansetron, or saline as prophylactic antiemetic therapy in patients at high risk of postoperative nausea and vomiting. Eur J Anaesthesiol. 2006;23(37):158.

45. Lee Y, Huang, P.K., Yang, Y.L., Lai, H.Y., Wang, J.J. A comparative study of haloperidol or dexamethasone plus ondansetron as prophylactic antiemetic therapy in patients at high risk of postoperative nausea and vomiting. Eur J Anaesthesiol. 2007;24(39):121.

46. Lipka F, Wappler, F., Scholz, J., Hans-Juergen Hennes, H.-J., Schulte am Esch, J. Antiemetic prophylaxis with an oral combination of tropisetron and dexamethasone by patients with anamnesis in PONV. Anesthesiology. 2002;96:A1193.

47. Lipka F, Wappler, F., Elßel, N., Scholz, J., Schulte am Esch, J. Antiemetic prophylaxis with an oral administration of tropisetron by patients with altered body habitus and a history of PONV. Eur J Anaesthesiol. 2004;21(32):17.

48. Meyer TA, Miltenburg, D., McAllister, R.K., Roberson, C.R. A comparison of combination antiemetic regimens for prevention of PONV in breast surgery patients (Group I: aprepitant 40 mg oral, ondansetron 4 mg IV and dexamethasone 4 mg IV vs. Group II: aprepitant 40 mg oral and dexamethasone 4 mg IV). Proceedings of the 2011 Annual Meeting of the American Society of Anesthesiologists. 2011:A850.

49. Mihailidis M, Macheridou, A., Kalantzi, N., Violari, M., Michaloliakou, C. Comparison of the effectiveness of preventive ondansetron monotherapy and ondansetron-dexamethasone combination in surgical patients at high risk for postoperative nausea and vomiting (PONV). Eur J Anaesthesiol. 2010;27(47):11-2.

50. Miles PJ, Ball, D.J., Melchoir, W.R., Dosch, M.P., Hurt, P.D. Is ondansetron as effective as droperidol in prevention of postoperative nausea and vomiting? AANA J. 1996;64(5):445.

51. Mohan VK, Darlong, V., Bodh, A.S., Kashyap, L. Prophylactic oral or intravenous ondansetron in prevention of postoperative nausea and vomiting in patients undergoing laparoscopic cholecystectomy. Anesthesiology. 2002;96:A1197.

52. Mostafa R, Wagieh, O., Awad, M.W., Al Kady, H. A comparison of the combination of granisetron and dexamethasone versus the combination of ondansetron and dexamethasone for the prevention of postoperative nausea and vomiting in patients undergoing spinal disc surgery. Eur J Anaesthesiol. 2016;33(e-Suppl 54):40-1.

53. Oh BJ, Jung, J.-W. Effect of total intravenous anesthesia and prophylactic 5-HT3 receptor antagonist on postoperative nausea and vomiting after gynecologic laparoscopic surgery: a prospective, randomized controlled study. Eur J Anaesthesiol. 2017;34(e-Suppl 55):2017.

54. Pacella E, Collini, C., Abdolrahimzadeh, B., Pacella, F., Brauneis, S., Gabrieli, C.B. The prophylaxis of emesis in emergency ophthalmic surgery: the use of ondansetron versus droperidol or metoclopramide. Invest Ophthalmol Vis Sci. 2001;42:165.

55. Pelavski A, Conesa, A., Gracia, C., Lopez Gil, V., García, Ll., Ruiz, P., Muñoz, C., Munar, F., Cahisa, M., Herbas, C. Low dose dexamethasone vs. ondansetron for antiemetic prophylaxis in breast surgery. Eur J Anaesthesiol. 2005;22(34):137-8.

56. Poeira R, Antunes, I., Filipe, H., Fernandes, A., Matos, F. Droperidol versus dexamethasone for postoperative nausea-vomiting prophylaxis in ophthalmic surgery. Proceedings of the 2010 Annual Meeting of the American Society of Anesthesiologists. 2010:A429.

57. Prabhu A, Venkatraghavan, L., Assmann, M., See, J., Tymianski, M. Prophylactic antiemetics do not reduce the incidence of postoperative nausea & vomiting in microvascular decompressions (MCD) of the trigeminal nerve. Eur J Anaesthesiol. 2006;23(37):106-7.

58. Radovanovic D, Milosev, S., Simic, M., Skoric-Jokic, S., Radovanovic, Z., Manic, D. Preoperative use of ondansetron and dexamethasone in thyroid gland surgery. Eur J Anaesthesiol. 2015;32(e-Suppl 53):64-5.

59. Rakanovic D, Sobot Novakovic, S., Svraka, D., Golic, D., Tomic, L., Grbavac, E. Antiemetic efficasy of TIVA and droperidol in laparoscopic cholecystectomy. Eur J Anaesthesiol. 2016;33(e-Suppl 54):57.

60. Ramesh M, Kamalakannan, P. Role of dexamethasone in acute postoperative pain, nausea and vomiting in patients undergoing laparoscopic gynecological surgeries as day care procedures. Anaesth Intensive Care 2011;39(4):725-6.

61. Roberson CR, Meyer, T.A., Davis, J., Rajab, M.H., Baisden, C.E. Randomized comparison of prophylactic ondansetron and dolasetron in the prevention of PONV in laparoscopic surgery. Anesthesiology. 2001;95:A19.

62. Ruesch D, Eberhart, L., Biedler, A., Dethling, J., Apfel, C.C. Randomized, risk-adapted comparison of ondansetron versus ondansetron plus dexamethasone to prevent post-operative nausea and vomiting. Anesthesiology. 2005;103:A625.

63. Shin YS, Kim, A. Comparison of palonosetron with ramosetron in prevention of postoperative nausea and vomiting in patients undergoing gynecological laparoscopic surgery and receiving postoperative intravenous patient-controlled analgesia. Eur J Anaesthesiol. 2011;28:139.

64. Silverberg MB, Pothula, V.R., Schiavone, A., Muddasani, P., Madupu, A., Allanku, S. Aprepitant for the prevention of postoperative nausea and vomiting: a prospective, randomized, double-blind, placebo-controlled study in patients undergoing laparoscopic cholecystectomy. Anesth Analg. 2012;114(5 Suppl 1):S-04.

65. Singh S. Palonosetron-dexamethasone combination for prophylaxis of post-operative nausea and vomiting after laparoscopic cholecystectomy. Anesth Analg. 2016;122(5 Suppl 3):S-288.

66. Siva Rama Krishna C, Babu, K.R., Murthy, K.S. A comparative study of ondansetron and palonosetron in post operative nausea and vomiting. Indian J Pharmacol. 2013;45:S71.

67. Soon Im K, Se Jin, L. The effect of aprepitant for the prevention of postoperative nausea and vomiting in patients undergoing gynecologic surgery with intravenous patient-controlled analgesia using fentanyl. Anesth Analg. 2012;1:S466.

68. Sousa A, Grigio, T.R., Degrande Pereira, M., Magalhaes Nunes Guimaraes, G., Ashmawi Adel, H. Aprepitant as prophylactic antiemetic therapy for postoperative nausea and vomiting after mastectomy in cancer patients: preliminary results of a RCT. Eur J Anaesthesiol. 2015;32(e-Suppl 53):266.

69. Spadafora SM, Moote, C.A. Postoperative nausea and vomiting after outpatient laparoscopy - a random doubleblind placebo comparison of ondansetron, droperidol and metoclopramide. Anesth Analg. 1994;78(2):U219.

70. Sun R, Klein, K., Skrivanek, G., White, P.F. Postoperative nausea and vomiting: sevoflurane vs desflurane. Anesth Analg. 1997;84:S552.

71. Tan CH, Onsiong, M.K., Kua, J.S.W. A comparison of droperidol and metoclopramide for the prevention of postoperative nausea and vomiting (PONV) associated with patient-controlled analgesia (PCA) morphine. Anaesth Intensive Care. 1998;26(3):324-5.

72. Tavlan A, Baltaci, B., Alptekin, A., Ceyhan, A., Unal, N. A comparison of the effect of single dose ondansetron and P6 (neiguan) acupuncture point on post-operative nausea and vomiting for gynaecological laparoscopy. Eur J Anaesthesiol. 1996;13:164.

73. TerRiet MF, Jacobs, J.S., Nunez, C.M., Gold, M.I. Dose-response effect of three antiemetics on alfentanil-related postoperative nausea and vomiting (PONV). Anesthesiology. 1997;87:A47.

74. Tigga R, Phukan, D. A comparative study of granisetron and ondansetron for prevention of nausea and vomiting following elective open abdominal surgeries under general anaesthesia in Silchar Medical College and Hospital, Silchar. Indian J Pharmacol. 2014;1:S23.

75. Warde GB, O'Connor, M., Dillane, D., Blunnie, W.P. "Balanced" antiemetic prophylaxis in patients undergoing gynaecological laparoscopy - single/double or triple therapy? Br J Anaesth. 1995;74(Suppl 1):7-8.

76. Zajac K, Zajac, M. Volatile anesthetics and prophylaxis of postoperative nausea and vomiting. Eur J Anaesthesiol. 2004;21(32):5.

77. Zarate E, Guevara, J., Botero, J., Rincon, D., Alarcon, I., Moreno, I., Marroquin, M. Evaluation of haloperidol dose needed to achieve complete control of postoperative nausea and vomiting. Anesthesiology. 2004;101:1454-63.
