# Supplementary material for: Include or not to include conference abstracts in systematic reviews? Lessons learned from a large Cochrane network meta-analysis including 585 trials
Source: Syst Rev. 2022 Aug 26;11:178. doi: 10.1186/s13643-022-02048-6 (PMC9413929; doi:10.1186/s13643-022-02048-6)
Supplement: Supplementary file 3 — Additional file 3. Study characteristics of eligible abstracts, abstracts awaiting classification and included studies in the review; data sheet with the study characteristics of eligible abstracts, abstracts awaiting classification and included studies in the review. [file 13643_2022_2048_MOESM3_ESM.docx]

**Supplementary File 3** Study characteristics of eligible abstracts, abstracts awaiting classification and included studies in the review

| Characteristics | Eligible abstracts  (n = 13) | Abstracts awaiting classification  (n = 77) | Studies included in the Cochrane review (n = 585) |
| --- | --- | --- | --- |
| Literature source | | | |
| excluded studies (Cochrane review; Weibel et al.) | 6 (46%) | 35 (45%) | - |
| ESA | 1 (8%) | 25 (32%) | - |
| ASA | 3 (23%) | 11 (14%) | - |
| IARS | 0 (0%) | 0 (0%) | - |
| Carlisle Review | 3 (23%) | 6 (8%) | - |
| Publication year | | | |
| 1965 - 1999 | 4 (31%) | 21 (27%) | 167 (29%) |
| 2000 - 2007 | 4 (31%) | 22 (29%) | 181 (31%) |
| 2008 - 2017 (until 6/11/2017) | 5 (38%) | 34 (44%) | 237 (41%) |
| Publication language | | | |
| English | 13 (100%) | 76 (99%) | 502 (86%) |
| others | 0 (0%) | 1 (1%) | 83 (14%) |
| Study conduct/origin of authors | | | |
| North America | 6 (46%) | 17 (22%) | 96 (16%) |
| Asia | 3 (23%) | 23 (30%) | 298 (51%) |
| Europe | 3 (23%) | 29 (38%) | 148 (25%) |
| South America | 1 (8%) | 3 (4%) | 11 (2%) |
| Africa | 0 (0%) | 3 (4%) | 9 (2%) |
| Australia/Oceania | 0 (0%) | 0 (0%) | 7 (1%) |
| no information provided | 0 (0%) | 2 (3%) | 0 (0%) |
| Setting | | | |
| multi-centre | 1 (8%) | 1 (1%) | 60 (10%) |
| single centre | 0 (0%) | 5 (6%) | 489 (84%) |
| no information provided | 12 (92%) | 71 (92%) | 36 (6%) |
| Funding | | | |
| by industry | 0 (0%) | 1 (1%) | 97 (17%) |
| not by industry | 1 (8%) | 3 (4%) | 104(18%) |
| no information provided | 12 (92%) | 73 (95%) | 384 (66%) |
| Study registration number | | | |
| provided | 0 (0%) | 1 (1%) | 75 (13%) |
| no information provided | 13 (100%) | 76 (99%) | 510 (87 %) |
| Study population | | | |
| median sample size (IQR [range]) (randomised participants) | 92 (60–135 [38–381]) | 90 (70–150 [34–5700] | 100 (70–160 [20–5199]) |
| mean age (SD) | 41 (2.5) | 49 (5.9) | 42 (12.5) |
| women | 49% | 53% | 83% |
| ASA I-II | 8 (62%) | 31 (40%) | 412 (70%) |
| received perioperative opioids | 2 (15%) | 29 (38%) | 514 (88%) |
|  |  |  |  |
| type of anaesthesia |  |  |  |
| inhalational anaesthesia | 2 (15%) | 24 (31%) | 515 (88%) |
| TIVA | 1 (8%) | 4 (5%) | 56 (10%) |
| both inhalational anaesthesia and TIVA | 0 (0%) | 1 (1%) | 6 (1%) |
| no information provided | 10 (77%) | 48 (62%) | 8 (1%) |
|  |  |  |  |
| type of surgery |  |  |  |
| gynaecological procedures | 2 (15%) | 17 (22%) | 189 (32%) |
| gastrointestinal procedures | 1 (8%) | 7 (9%) | 112 (19%) |
| neurological procedures | 3 (23%) | 3 (4%) | 13 (2%) |
| any other or not reported | 7 (54%) | 50 (65%) | 271 (46%) |
| Intervention |  |  |  |
| inactive control arm (placebo or no intervention) | 5 (38%) | 39 (51%) | 384 (66%) |
| only active arms | 8 (62 %) | 38 (49%) | 201 (34%) |
|  |  |  |  |
| only single interventions | 8 (62%) | 48 (62%) | 423 (72%) |
| only combination interventions | 1 (8%) | 10 (13%) | 31 (5%) |
| both single and combination interventions | 4 (31 %) | 19 (25%) | 131 (23%) |
