# Supplementary material for: Include or not to include conference abstracts in systematic reviews? Lessons learned from a large Cochrane network meta-analysis including 585 trials
Source: Syst Rev. 2022 Aug 26;11:178. doi: 10.1186/s13643-022-02048-6 (PMC9413929; doi:10.1186/s13643-022-02048-6)
Supplement: Supplementary file 4 — Additional file 4. Reporting of data for data extraction according to the Cochrane review’s extraction sheet (25 items); data sheet with the assessment of reporting quality of all abstracts regarding data extraction items. [file 13643_2022_2048_MOESM4_ESM.docx]

**Supplementary File 4** Reporting of data for data extraction according to the Cochrane review´s extraction sheet (25 items)

| Items | Sufficient information (n = 90) | Insufficient information (n = 90) | Missing  Information (n = 90) | Insufficient/missing information in abstracts before 2008 (n = 51) | Insufficient/missing information in abstracts since 2008 (n = 39) |
| --- | --- | --- | --- | --- | --- |
| Study identification | | | | | |
| Sponsorship source | 5 (6%) | 0 (0%) | 85 (94%) | 49 (96%) | 36 (92%) |
| Setting | 26 (29%) | 0 (0%) | 64 (71%) | 35 (69%) | 29 (74%) |
| Institution | 87 (97%) | 1 (1%) | 2 (2%) | 2 (4%) | 1 (3%) |
| E-mail address | 1 (1%) | 0 (0%) | 89 (99%) | 50 (98%) | 39 (100%) |
| Duration of study | 5 (6%) | 0 (0%) | 85 (94%) | 50 (98%) | 35 (90%) |
| Study’s primary outcome | 5 (6%) | 0 (0%) | 85 (94%) | 48 (94%) | 37 (95%) |
| Trial registry number | 1 (1%) | 0 (0%) | 89 (99%) | 51 (100%) | 38 (97%) |
| Study design | | | | | |
| Study design (RCT) | 84 (93%) | 6 (7%) | 0 (0%) | 5 (10%) | 1 (3%) |
| Population | | | | | |
| Inclusion criteria | 77 (86%) | 13 (14%) | 0 (0%) | 6 (12%) | 7 (18%) |
| Exclusion criteria | 9 (10%) | 0 (0%) | 81 (90%) | 45 (88%) | 36 (92%) |
| Number enrolled, randomised and analysed | 21 (23%) | 69 (77%) | 0 (0%) | 39 (76%) | 30 (77%) |
| Basic characteristics |  |  |  |  |  |
| Age | 34 (38%) | 16 (18%) | 40 (44%) | 32 (63%) | 24 (62%) |
| Weight or BMI | 14 (16%) | 0 (0%) | 76 (84%) | 43 (84%) | 33 (85%) |
| ASA classification | 38 (42%) | 9 (10%) | 43 (47%) | 24 (47%) | 28 (72%) |
| Apfel risk factors |  |  |  |  |  |
| History of PONV/motion sickness | 9 (10%) | 5 (6%) | 76 (84%) | 43 (84%) | 38 (97%) |
| Smoking status | 4 (4%) | 4 (4%) | 82 (91%) | 49 (96%) | 37 (95%) |
| Gender | 42 (47%) | 3 (3%) | 45 (50%) | 23 (45%) | 25 (64%) |
| Perioperative opioids | 31 (34%) | 2 (2%) | 57 (63%) | 30 (59%) | 29 (74%) |
| Type of surgery | 78 (87%) | 3 (3%) | 9 (10%) | 7 (14%) | 5 (13 %) |
| Duration of anaesthesia | 9 (10%) | 0 (0%) | 81 (90%) | 44 (86%) | 37 (95%) |
| Intervention | | | | | |
| Dose | 87 (97%) | 0 (0%) | 3 (3%) | 1 (2%) | 2 (5 %) |
| Time point of administration | 73 (81%) | 7 (8%) | 10 (11%) | 5 (10%) | 12 (31%) |
| Route of administration | 60 (67%) | 2 (2%) | 28 (31%) | 16 (31%) | 14 (36%) |
| Rescue antiemetics | 17 (19%) | 19 (21%) | 54 (60%) | 42 (82%) | 31 (79%) |
| Outcomes | | | | | |
| Outcome details and data | 19 (21%) | 71 (79%) | 0 (0%) | 37 (73 %) | 34 (87%) |

**Definition of items**

| Items | Definition and explanation |
| --- | --- |
| Study identification |  |
| Sponsorship source | Reporting of a specific source of funding was assessed sufficient. |
| Setting | The setting includes information about the number of study centres involved, and whether inpatient or outpatient surgery is investigated. The reporting of a specific hospital or department is considered “sufficient”, too. |
| Institution | Reporting of the institution authors belong to, is considered sufficient. It is assessed “insufficient” if the study location but not the institution is available. |
| E-mail address | Information on the e-mail address of the corresponding author was assessed sufficient. |
| Duration of study | Information on the time frame when the study was conducted, was assessed sufficient. |
| Study’s primary outcome | Authors should explicitly state the primary outcome for the trial. No credit should be given for an outcome that is not defined as primary. Therefore, this is considered “missing information”. |
| Trial registry number | Authors should report the trial registry number. If it is stated that the trial had not been registered, this information is assessed as sufficient, too. |
|  |  |
| Study design | The study needs to be identified as randomized controlled trial. Also, the number of study arms should be stated. If the study is not explicitly randomized, this is considered insufficient. |
|  |  |
| Population |  |
| Inclusion criteria | The reporting of more than one inclusion criteria was assessed as sufficient, one inclusion criteria only as insufficient. |
| Exclusion criteria | The reporting of at least one exclusion criteria was assessed as sufficient. |
| Number enrolled, randomized and analysed | This item was assessed as sufficient when at least both the overall numbers randomized and analysed were available. Less precise phrases (e.g. enrolled – “assigned” in combination with analysed) were considered sufficient, too. When none of these numbers was reported, the information was considered missing. |
| Basic characteristics | When age, weight or BMI, and ASA classification were all sufficiently reported, basic characteristics were assessed as sufficient. If none of them are reported, basic characteristics were assessed as missing information. |
| Age | Reporting of age as mean, or range was considered sufficient. Information per study arm would be even more informative. The phrase “adults” is considered insufficient, as depending on the cultural background interpretations can differ. The reporting of “men” or “women” is assessed as “missing information” regarding age. |
| Weight or BMI | Reporting of weight or BMI as mean, or range is considered sufficient. Information per study arm would be even more informative. |
| ASA classification | Authors should report ASA classification of participants. The phrase “healthy participants” is assessed as insufficient information regarding ASA classification. |
| Apfel risk factors | When history of PONV/motion sickness, smoking status, gender and perioperative opioids were all sufficiently reported, Apfel risk factors were assessed as sufficient overall. If none of them are reported, Apfel risk factors overall were assessed as missing information. |
| History of PONV/motion sickness | Information on history of PONV/motion sickness was assessed sufficient when corresponding information were available. |
| Smoking status | Information on smoking status was assessed sufficient when corresponding information were available. |
| Gender | Reporting of gender was assessed sufficient when information (e.g. as inclusion criteria or proportion of women) were available. |
| Perioperative opioids | Reporting of perioperative opioids was assessed sufficient when the application or the missing application of opioids was stated. |
| Type of surgery | Information on the type of surgery was assessed sufficient when information regarding the speciality field or specific surgery were available. Phrases like “outpatient surgery” or information that did not apply to all study arms were considered insufficient. |
| Duration of anaesthesia | Information on duration of anaesthesia (or alternatively duration of surgery) as mean or range was assessed as sufficient. The information per study arm would be even more informative. |
|  |  |
| Intervention |  |
| Dose | Reporting of dose was assessed sufficient when a dose was available per study arm. |
| Time point of administration | Information on time point of administration was assessed sufficient when a time point was available per active study arm. Imprecise phrases (e.g. “prophylactic”) were considered insufficient as well as time points not available for each study arm. |
| Route of administration | Information on route of administration was assessed sufficient when information regarding route was available per active study arm. Routes not available for each study arm were considered insufficient. |
| Rescue antiemetics | Reporting of rescue antiemetics was assessed sufficient if the applied rescue antiemetic was stated (no dose required as we focused on prevention of PONV). Mentioning rescue antiemetics without naming the applied antiemetic was considered insufficient. |
| Outcomes |  |
| Outcome details | Reporting of outcome details was assessed sufficient when in each study arm the number or the percentage of participants with or without the event and the number analysed were stated. When no number analysed or only qualitative descriptions (e.g. “similar”, “(no) significant difference”) were available, outcome details were assessed insufficient. When only one of multiple outcomes was assessed sufficient, overall information was assessed insufficient. |
