# Supplementary material for: Include or not to include conference abstracts in systematic reviews? Lessons learned from a large Cochrane network meta-analysis including 585 trials
Source: Syst Rev. 2022 Aug 26;11:178. doi: 10.1186/s13643-022-02048-6 (PMC9413929; doi:10.1186/s13643-022-02048-6)
Supplement: Supplementary file 5 — Additional file 5. Reporting of information for risk of bias assessment in abstracts; data sheet with the assessment of reporting quality of all abstracts regarding risk of bias relevant information. [file 13643_2022_2048_MOESM5_ESM.docx]

**Supplementary File 5** Reporting of information for risk of bias assessment in abstracts

|  | Sufficient information (n = 90) | Insufficient information (n = 90) | Missing  Information (n = 90) | Insufficient/missing information in abstracts before 2008 (n = 51) | Insufficient/missing information in abstracts since 2008 (n = 39) |
| --- | --- | --- | --- | --- | --- |
| Random sequence generation | 2 (2%) | 87 (97%) | 1 (1%) | 49 (96%) | 39 (100%) |
| Allocation concealment | 1 (1%) | 3 (3%) | 86 (96%) | 50 (98%) | 39 (100%) |
| Blinding of participants and personnel | 2 (2%) | 55 (61%) | 33 (37%) | 50 (98%) | 38 (97%) |
| Blinding of outcome assessment | 7 (8%) | 0 (0%) | 83 (92%) | 47 (92%) | 36 (92%) |
| Incomplete outcome data | 34 (38%) | 56 (62%) | 0 (0%) | 32 (63%) | 24 (62%) |
| Selective reporting | 0 (0%) | 1 (1%) | 89 (99%) | 51 (100%) | 39 (100%) |
| Other bias | 0 (0%) | 58 (64%) | 32 (36%) | 51 (100%) | 39 (100%) |
