# Supplementary material for: Include or not to include conference abstracts in systematic reviews? Lessons learned from a large Cochrane network meta-analysis including 585 trials
Source: Syst Rev. 2022 Aug 26;11:178. doi: 10.1186/s13643-022-02048-6 (PMC9413929; doi:10.1186/s13643-022-02048-6)
Supplement: Supplementary file 6 — Additional file 6. Adherence to CONSORT for abstracts reporting items; data sheet with the assessment of reporting quality of all abstracts regarding the CONSORT for abstracts checklist. [file 13643_2022_2048_MOESM6_ESM.docx]

**Supplementary File 6** Adherence to CONSORT for Abstracts reporting items

| Items | Description | Sufficient information (n = 90) | Insufficient information (n = 90) | Missing information (n = 90) | Insufficient/missing information in abstracts before 2008 (n = 51) | Insufficient/missing information in abstracts since 2008 (n = 39) |
| --- | --- | --- | --- | --- | --- | --- |
| Title | Identification of the study as randomized | 14 (16%) | 0 (0%) | 76 (84%) | 45 (88%) | 31 (79%) |
| Authors | Contact details for the corresponding author | 0 (0%) | 1 (1%) | 89 (99%) | 51 (100%) | 39 (100%) |
| Trial Design | Description of the trial design (e.g. parallel, cluster, non-inferiority) | 84 (93%) | 6 (7%) | 0 (0%) | 5 (10%) | 1 (3%) |
| Methods | | | | | | |
| Participants | Eligibility criteria for participants and the settings where data were collected | 24 (27%) | 66 (73%) | 0 (0%) | 35 (69%) | 31 (79%) |
| Interventions | Interventions intended for each group | 51 (57%) | 38 (42%) | 1 (1%) | 17 (33%) | 22 (56%) |
| Objective | Specific objective or hypothesis | 30 (33%) | 55 (61%) | 5 (6%) | 38 (75%) | 22 (56%) |
| Outcome | Clearly defined primary outcome for this report | 4 (4%) | 1 (1%) | 85 (94%) | 49 (96%) | 37 (95%) |
| Randomization | How participants were allocated to interventions | 0 (0%) | 6 (7%) | 84 (93%) | 51 (100%) | 39 (100%) |
| Blinding (masking) | Whether or not participants, care givers, and those assessing the outcomes were blinded to group assignment | 2 (2%) | 58 (64%) | 30 (33%) | 50 (98%) | 38 (97%) |
| Results | | | | | | |
| Numbers randomized | Number of participants randomized to each group | 22 (24%) | 22 (24%) | 46 (51%) | 43 (84%) | 25 (64%) |
| Recruitment | Trial status | 4 (4%) | 6 (7%) | 80 (89%) | 51 (100%) | 35 (90%) |
| Numbers analyzed | Number of participants analysed in each group | 37 (41%) | 4 (4%) | 49 (54%) | 27 (53%) | 26 (67%) |
| Outcome | For the primary outcome, a result for each group and the estimated effect size and its precision | 0 (0%) | 81 (90%) | 9 (10%) | 51 (100%) | 39 (100%) |
| Harms | Important adverse events or side effects | 12 (13%) | 22 (24%) | 56 (62%) | 41 (80%) | 37 (95%) |
| Conclusions | General interpretation of the results | 90 (100%) | 0 (0%) | 0 (0%) | 0 (0%) | 0 (0%) |
| Trial registration | Registration number and name of trial register | 1 (1%) | 0 (0%) | 89 (99%) | 51 (100%) | 38 (97%) |
| Funding | Source of funding | 4 (4%) | 1 (1%) | 85 (94%) | 49 (96%) | 37 (95%) |
