# Supplementary material for: Include or not to include conference abstracts in systematic reviews? Lessons learned from a large Cochrane network meta-analysis including 585 trials
Source: Syst Rev. 2022 Aug 26;11:178. doi: 10.1186/s13643-022-02048-6 (PMC9413929; doi:10.1186/s13643-022-02048-6)
Supplement: Supplementary file 7 — Additional file 7. Effect estimates of antiemetic treatments in the abstracts and the NMA; data sheet comparing effect estimates of antiemetic treatments in the abstracts vs. review (direct evidence/NMA). [file 13643_2022_2048_MOESM7_ESM.docx]

**Additional File 7** Effect estimates of antiemetic treatments in the abstracts and the NMA

| Outcome | Eligible abstracts included in sensitivity NMA | Treatment comparison | Estimated treatment effect in abstract’s study results  RR (95% CI) and number of events/participants per group | Estimated treatment effect  RR (95% CI) in review  without abstracts | |
| --- | --- | --- | --- | --- | --- |
|  |  |  |  | NMA | Direct evidence |
| Vomiting 24h | Ilbeigi 1999 ^Ref. 5^ | meto vs. meto-onda | 11.00 (0.66, 182.87)  5/15 vs. 0/15 | 21.36  (1.30; 351.62) | 25.00  (1.50; 417.19) |
|  |  | meto vs. onda | 11.00 (0.66, 182.87)  5/15 vs. 0/15 | 1.33  (1.13; 1.56) | 1.55  (1.23; 1.96) |
|  |  | meto-onda vs. onda | Not estimable  0/15 vs. 0/15 | 0.06  (0.004; 1.02) | 0.09  (0.01; 1.62) |
|  | White 2005 ^Ref. 13^ | palo vs. plac | 0.65 (0.49, 0.86)  107/310 vs. 33/62 | 0.62  (0.48; 0.80) | 0.68  (0.51; 0.92) |
| Headache | Ilbeigi 1999 ^Ref. 5^ | meto vs. meto-onda | 0.33 (0.01, 7.58)  0/15 vs. 1/15 | 1.21  (0.29; 5.09) | 0.20  (0.01; 3.96) |
|  |  | meto vs. onda | 0.33 (0.01, 7.58)  0/15 vs. 1/15 | 0.80  (0.65; 0.97) | 0.84  (0.65; 1.08) |
|  |  | meto-onda vs. onda | 1.00 (0.07, 14.55)  1/15 vs. 1/15 | 0.66  (0.16; 2.73) | 0.56  (0.13; 2.36) |
|  | White 2005 ^Ref. 13^ | palo vs. plac | 1.36 (0.55, 3.34)  34/310 vs. 5/62 | 1.15  (0.89; 1.49) | 1.29  (0.87; 1.92) |
| QT pro-longation | White 2005 ^Ref. 13^ | palo vs. plac | Not estimable  0/310 vs. 0/62 | 0.16 (0.02; 1.39) | 0.11  (0.005; 2.68) |
| Vomiting „early“ | Meyer 2004 ^Ref. 7^ | dola vs. dola-scop | 1.08 (0.44, 2.62)  9/69 vs. 8/66 | NA | NA |
|  | Sun 1995 ^Ref. 12^ | drop vs. drop-meto | 1.20 (0.42, 3.43)  6/25 vs. 5/25 | 1.80  (0.55; 5.89) | 1.58  (0.34; 7.33) |
|  |  | drop vs. meto | 0.67 (0.28, 1.59)  6/25 vs. 9/25 | 0.54  (0.42; 0.69) | 0.57  (0.36; 0.91) |
|  |  | drop vs. onda | 3.00 (0.67, 13.46) 6/25 vs. 2/25 | 0.99  (0.80; 1.23) | 0.83  (0.56; 1.23) |
|  |  | drop vs. plac | 1.20 (0.42, 3.43)  6/25 vs. 5/25 | 0.44  (0.36; 0.53) | 0.46  (0.37; 0.57) |
|  |  | drop-meto vs. meto | 0.56 (0.22, 1.43)  5/25 vs. 9/25 | 0.30  (0.09; 0.98) | 0.23  (0.05; 1.05) |
|  |  | drop-meto vs. onda | 2.50 (0.53, 11.70)  5/25 vs. 2/25 | 0.55  (0.19; 1.79) | NA |
|  |  | drop-meto vs. plac | 1.00 (0.33, 3.03)  5/25 vs. 5/25 | 0.24  (0.08; 0.80) | 0.14  (0.03; 0.62) |
|  |  | meto vs. onda | 4.50 (1.08, 18.77)  9/25 vs. 2/25 | 1.83  (1.49; 2.24) | 1.94  (1.33; 2.82) |
|  |  | meto vs. plac | 1.80 (0.70, 4.62)  9/25 vs. 5/25 | 0.82 (0.68; 0.98) | 0.81  (0.64; 1.01) |
|  |  | onda vs. plac | 0.40 (0.09, 1.87)  2/25 vs. 5/25 | 0.45  (0.39; 0.50) | 0.46  (0.40; 0.53) |
| Vomiting „late“ | Meyer 2004 ^Ref. 7^ | dola vs. dola-scop | 2.23 (0.91, 5.46)  14/69 vs. 6/66 | NA | NA |
| Nausea | George 2016 ^Ref. 2^ | dime vs. plac | 0.75 (0.43, 1.31)  18/72 vs. 17/51 | 0.51  (0.30; 0.88) | 0.33  (0.13; 0.88) |
|  | Ilbeigi 1999 ^Ref. 5^ | meto vs. meto-onda | 5.00 (0.66, 37.85)  5/15 vs. 1/15 | 9.67  (1.32; 71.03) | 10.00  (1.31; 76.11) |
|  |  | meto vs. onda | 2.50 (0.57, 10.93)  5/15 vs. 2/15 | 1.16  (1.03; 1.30) | 1.22  (1.02; 1.45) |
|  |  | meto-onda vs. onda | 0.50 (0.05, 4.94)  1/15 vs. 2/15 | 0.12  (0.02; 0.88) | 0.13  (0.02; 0.97) |
|  | Samra 2003 ^Ref. 9^ | dola vs. drop | 0.93 (0.70, 1.23)  38/65 vs. 39/62 | 1.25 (1.03; 1.53) | 1.18 (0.83; 1.68) |
|  |  | dola vs. onda | 1.13 (0.82, 1.56)  38/65 vs. 31/60 | 1.08 (0.89; 1.30) | 1.14 (0.78; 1.64) |
|  |  | dola vs. plac | 1.02 (0.76, 1.38)  38/65 vs. 36/63 | 0.74 (0.62; 0.90) | 0.77 (0.60; 0.99) |
|  |  | drop vs. onda | 1.22 (0.89, 1.66)  39/62 vs. 31/60 | 0.86  (0.77; 0.96) | 0.96  (0.79; 1.17) |
|  |  | drop vs. plac | 1.10 (0.83, 1.47)  39/62 vs. 36/63 | 0.59  (0.54; 0.65) | 0.59  (0.53; 0.66) |
|  |  | onda vs. plac | 0.90 (0.65, 1.25)  31/60 vs. 36/63 | 0.69 (0.65; 0.74) | 0.69  (0.64; 0.74) |
| Complete response | Samra 2003 ^Ref. 9^ | dola vs. drop | 0.89 (0.58, 1.38)  27/65 vs. 23/62 | 0.97 (0.85; 1.11) | 0.95 (0.75; 1.20) |
|  |  | dola vs. onda | 0.86 (0.58, 1.27)  27/65 vs. 29/60 | 0.97  (0.85; 1.11) | 0.95  (0.75; 1.20) |
|  |  | dola vs. plac | 0.97 (0.65, 1.45)  27/65 vs. 27/63 | 1.45  (1.29; 1.64) | 1.45  (1.26; 1.66) |
|  |  | drop vs. onda | 0.77 (0.51, 1.16) 23/62 vs. 29/60 | 1.00  (0.91; 1.10) | 1.09  (0.94; 1.27) |
|  |  | drop vs. plac | 0.87 (0.56, 1.33)  23/62 vs. 27/63 | 1.50  (1.37; 1.64) | 1.42  (1.27; 1.58) |
|  |  | onda vs. plac | 1.13 (0.77, 1.66)  29/60 vs. 27/63 | 1.49  (1.40; 1.60) | 1.55  (1.42; 1.69) |
|  | White 2005 ^Ref. 13^ | palo vs. plac | 1.82 (1.07, 3.09)  109/310 vs. 12/62 | 1.62  (1.39; 1.88) | 1.41  (1.15; 1.72) |

References from Additional File 2, eligible abstracts

CI = confidence interval, NA= not applicable, Ref. = reference, RR = risk ratio, vs. = versus

dexa = dexamethasone, dime = dimenhydrinate, dola = dolasetron, drop= droperidol, meto = metoclopramide, onda = ondansetron, palo = palonosetron, plac = placebo, scop = scopolamine
