# Supplementary material for: Include or not to include conference abstracts in systematic reviews? Lessons learned from a large Cochrane network meta-analysis including 585 trials
Source: Syst Rev. 2022 Aug 26;11:178. doi: 10.1186/s13643-022-02048-6 (PMC9413929; doi:10.1186/s13643-022-02048-6)
Supplement: Supplementary file 8 — Additional file 8. Forest plots NMA; Sensitivity analyses including abstracts; graphical presentation of the NMA effect estimates of treatments per outcome with and without abstracts. [file 13643_2022_2048_MOESM8_ESM.pdf]

**Supplementary File 8** Forest Plots NMA; Sensitivity analyses including abstracts

Outcome: Vomiting within 24 hours (without abstracts)

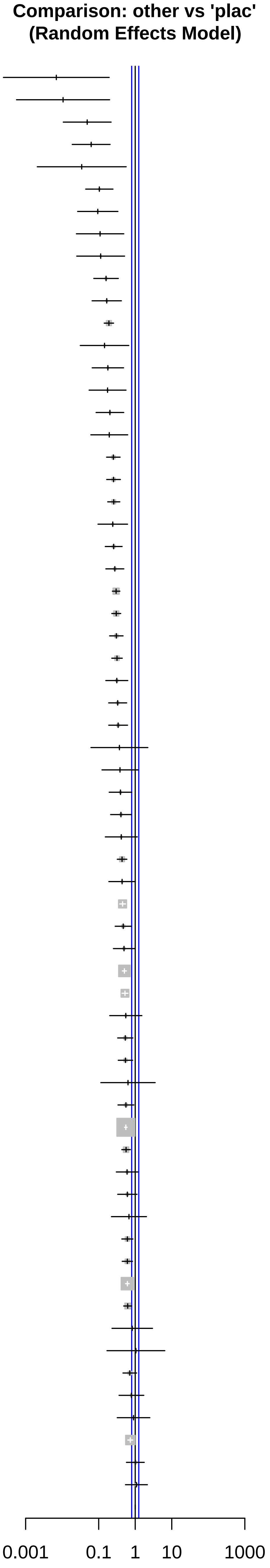


| Treatment | RR | 95%−CI |
|---|---|---|
| apre−palo | 0.01 | [0.00; 0.19] |
| dexa−meto−onda | 0.01 | [0.00; 0.20] |
| apre−ramo | 0.05 | [0.01; 0.22] |
| fosa | 0.06 | [0.02; 0.21] |
| meto−onda | 0.03 | [0.00; 0.57] |
| apre−dexa | 0.10 | [0.04; 0.25] |
| dexa−dola | 0.09 | [0.03; 0.34] |
| dexa−drop−meto−onda | 0.11 | [0.02; 0.49] |
| cp12−onda | 0.11 | [0.03; 0.51] |
| caso | 0.16 | [0.07; 0.35] |
| apre−onda | 0.17 | [0.07; 0.42] |
| caso−onda | 0.19 | [0.14; 0.26] |
| dexa−ramo | 0.14 | [0.03; 0.67] |
| apre−dexa−onda | 0.18 | [0.07; 0.48] |
| pene−trop | 0.17 | [0.05; 0.56] |
| halo−onda | 0.20 | [0.08; 0.49] |
| meth−trop | 0.19 | [0.06; 0.62] |
| dexa−drop−onda | 0.25 | [0.16; 0.39] |
| dexa−gran | 0.25 | [0.17; 0.39] |
| apre | 0.26 | [0.18; 0.38] |
| onda−prom | 0.24 | [0.10; 0.62] |
| dexa−trop | 0.26 | [0.15; 0.44] |
| dexa−palo | 0.28 | [0.16; 0.49] |
| dexa−onda | 0.30 | [0.24; 0.38] |
| drop−onda | 0.30 | [0.23; 0.40] |
| dexa−halo | 0.30 | [0.20; 0.46] |
| dexa−drop | 0.32 | [0.23; 0.44] |
| dola−drop | 0.31 | [0.16; 0.63] |
| onda−vest | 0.33 | [0.19; 0.58] |
| drop−gran | 0.34 | [0.19; 0.62] |
| mecl−onda | 0.37 | [0.06; 2.22] |
| dexa−scop | 0.38 | [0.12; 1.19] |
| cp12 | 0.39 | [0.19; 0.80] |
| drop−trop | 0.41 | [0.21; 0.78] |
| drop−palo | 0.42 | [0.15; 1.14] |
| ramo | 0.44 | [0.32; 0.59] |
| dexa−prom | 0.44 | [0.19; 1.01] |
| gran | 0.45 | [0.38; 0.54] |
| onda−scop | 0.47 | [0.28; 0.78] |
| aliz | 0.49 | [0.25; 0.96] |
| dexa | 0.51 | [0.44; 0.57] |
| trop | 0.52 | [0.43; 0.63] |
| dime | 0.55 | [0.20; 1.52] |
| prom | 0.53 | [0.33; 0.86] |
| dexa−meto | 0.54 | [0.34; 0.85] |
| apre−scop | 0.63 | [0.11; 3.52] |
| rola | 0.56 | [0.34; 0.92] |
| onda | 0.55 | [0.51; 0.60] |
| dola | 0.56 | [0.43; 0.74] |
| pred | 0.60 | [0.30; 1.18] |
| proc | 0.61 | [0.33; 1.12] |
| dixy | 0.67 | [0.22; 2.03] |
| halo | 0.61 | [0.43; 0.87] |
| scop | 0.61 | [0.44; 0.84] |
| drop | 0.61 | [0.54; 0.69] |
| palo | 0.62 | [0.48; 0.80] |
| cycl | 0.83 | [0.23; 2.98] |
| meto−prom | 1.04 | [0.17; 6.45] |
| amis | 0.71 | [0.46; 1.09] |
| pene | 0.79 | [0.36; 1.72] |
| beta | 0.90 | [0.32; 2.51] |
| meto | 0.73 | [0.63; 0.86] |
| domp | 1.01 | [0.57; 1.77] |
| busp | 1.08 | [0.54; 2.15] |
| plac | 1.00 | |

Outcome: Vomiting within 24 hours (including abstracts)

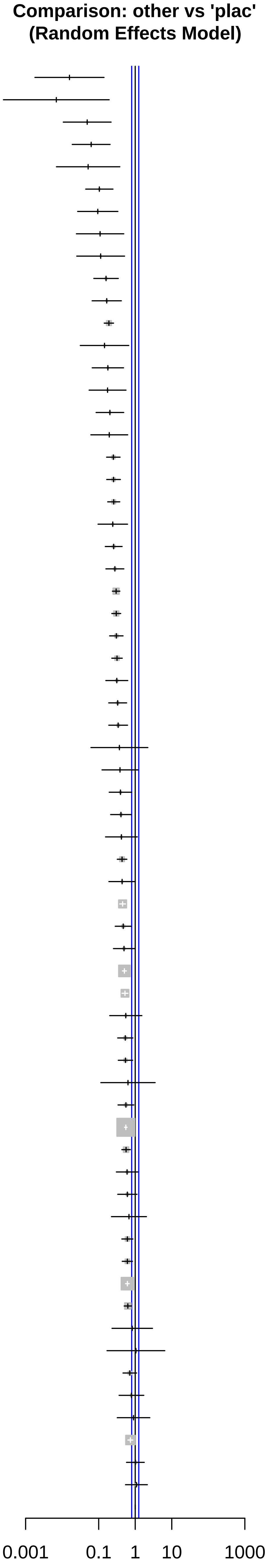


| Treatment | RR | 95%−CI |
|---|---|---|
| dexa−meto−onda | 0.02 | [0.00; 0.14] |
| apre−palo | 0.01 | [0.00; 0.19] |
| apre−ramo | 0.05 | [0.01; 0.22] |
| fosa | 0.06 | [0.02; 0.21] |
| meto−onda | 0.05 | [0.01; 0.38] |
| apre−dexa | 0.10 | [0.04; 0.25] |
| dexa−dola | 0.09 | [0.03; 0.34] |
| dexa−drop−meto−onda | 0.11 | [0.02; 0.49] |
| cp12−onda | 0.11 | [0.03; 0.51] |
| caso | 0.16 | [0.07; 0.34] |
| apre−onda | 0.17 | [0.07; 0.42] |
| caso−onda | 0.19 | [0.14; 0.26] |
| dexa−ramo | 0.14 | [0.03; 0.67] |
| apre−dexa−onda | 0.18 | [0.07; 0.48] |
| pene−trop | 0.17 | [0.05; 0.56] |
| halo−onda | 0.20 | [0.08; 0.48] |
| meth−trop | 0.19 | [0.06; 0.62] |
| dexa−drop−onda | 0.25 | [0.16; 0.39] |
| dexa−gran | 0.26 | [0.17; 0.39] |
| apre | 0.26 | [0.18; 0.38] |
| onda−prom | 0.24 | [0.10; 0.62] |
| dexa−trop | 0.26 | [0.15; 0.44] |
| dexa−palo | 0.28 | [0.16; 0.49] |
| dexa−onda | 0.30 | [0.24; 0.38] |
| drop−onda | 0.30 | [0.23; 0.40] |
| dexa−halo | 0.30 | [0.20; 0.46] |
| dexa−drop | 0.32 | [0.23; 0.44] |
| dola−drop | 0.31 | [0.16; 0.63] |
| onda−vest | 0.33 | [0.19; 0.58] |
| drop−gran | 0.34 | [0.19; 0.62] |
| mecl−onda | 0.37 | [0.06; 2.22] |
| dexa−scop | 0.38 | [0.12; 1.19] |
| cp12 | 0.39 | [0.19; 0.80] |
| drop−trop | 0.41 | [0.21; 0.78] |
| drop−palo | 0.42 | [0.15; 1.14] |
| ramo | 0.44 | [0.32; 0.59] |
| dexa−prom | 0.44 | [0.19; 1.01] |
| gran | 0.45 | [0.38; 0.54] |
| onda−scop | 0.47 | [0.28; 0.78] |
| aliz | 0.49 | [0.25; 0.96] |
| dexa | 0.51 | [0.45; 0.57] |
| trop | 0.52 | [0.43; 0.63] |
| dime | 0.55 | [0.20; 1.52] |
| prom | 0.53 | [0.33; 0.86] |
| dexa−meto | 0.54 | [0.34; 0.85] |
| apre−scop | 0.63 | [0.11; 3.51] |
| rola | 0.56 | [0.34; 0.91] |
| onda | 0.55 | [0.51; 0.60] |
| dola | 0.56 | [0.43; 0.74] |
| pred | 0.60 | [0.30; 1.18] |
| proc | 0.61 | [0.33; 1.12] |
| dixy | 0.67 | [0.22; 2.03] |
| halo | 0.61 | [0.43; 0.87] |
| scop | 0.61 | [0.44; 0.84] |
| drop | 0.61 | [0.54; 0.69] |
| palo | 0.63 | [0.50; 0.79] |
| cycl | 0.83 | [0.23; 2.97] |
| meto−prom | 1.04 | [0.17; 6.45] |
| amis | 0.71 | [0.46; 1.09] |
| pene | 0.79 | [0.36; 1.72] |
| beta | 0.90 | [0.32; 2.51] |
| meto | 0.74 | [0.63; 0.86] |
| domp | 1.01 | [0.58; 1.77] |
| busp | 1.08 | [0.54; 2.15] |
| plac | 1.00 | |

Outcome: Headache (without abstracts)

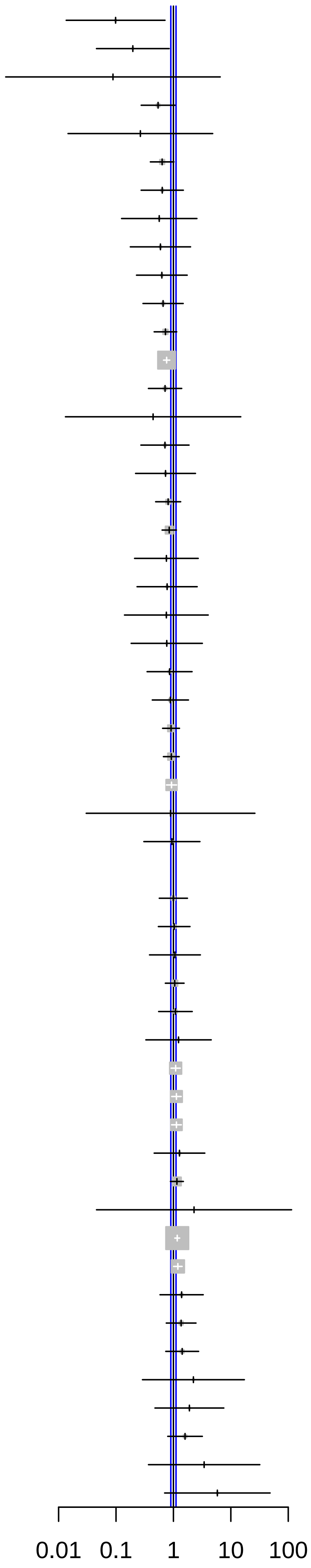


| Treatment | RR | 95%−CI |
|---|---|---|
| sulp | 0.10 | [0.01; 0.71] |
| drop−trop | 0.20 | [0.05; 0.84] |
| dexa−prom | 0.09 | [0.00; 6.54] |
| halo | 0.54 | [0.27; 1.06] |
| prom | 0.27 | [0.01; 4.83] |
| dola−drop | 0.64 | [0.40; 1.02] |
| pred | 0.64 | [0.27; 1.49] |
| dexa−meto−onda | 0.57 | [0.12; 2.56] |
| drop−onda | 0.59 | [0.18; 1.99] |
| meto−trop | 0.63 | [0.23; 1.74] |
| onda−scop | 0.66 | [0.29; 1.48] |
| dexa−gran | 0.73 | [0.46; 1.14] |
| drop | 0.76 | [0.67; 0.86] |
| halo−onda | 0.71 | [0.37; 1.39] |
| onda−prom | 0.44 | [0.01; 14.89] |
| drop−gran | 0.71 | [0.27; 1.87] |
| pene | 0.73 | [0.22; 2.42] |
| caso | 0.81 | [0.49; 1.34] |
| dexa | 0.85 | [0.63; 1.13] |
| dexa−scop | 0.75 | [0.21; 2.70] |
| azas | 0.78 | [0.23; 2.59] |
| apre−palo | 0.75 | [0.14; 4.03] |
| meto−onda | 0.77 | [0.18; 3.19] |
| apre | 0.86 | [0.35; 2.11] |
| dexa−drop | 0.88 | [0.43; 1.83] |
| caso−onda | 0.91 | [0.65; 1.27] |
| dexa−onda | 0.92 | [0.67; 1.26] |
| meto | 0.93 | [0.76; 1.13] |
| dexa−halo | 0.89 | [0.03; 26.26] |
| apre−ramo | 0.94 | [0.31; 2.89] |
| plac | 1.00 | |
| dexa−ramo | 1.00 | [0.57; 1.75] |
| scop | 1.03 | [0.55; 1.94] |
| onda−vest | 1.06 | [0.38; 2.95] |
| apre−dexa | 1.05 | [0.72; 1.53] |
| dexa−meto | 1.08 | [0.55; 2.13] |
| dime | 1.23 | [0.33; 4.55] |
| trop | 1.09 | [0.91; 1.32] |
| dola | 1.13 | [0.93; 1.36] |
| ramo | 1.13 | [0.93; 1.37] |
| pene−trop | 1.28 | [0.46; 3.52] |
| palo | 1.15 | [0.89; 1.49] |
| drop−palo | 2.29 | [0.05; 114.22] |
| onda | 1.16 | [1.06; 1.28] |
| gran | 1.20 | [1.01; 1.42] |
| dixy | 1.39 | [0.58; 3.31] |
| dexa−trop | 1.36 | [0.75; 2.47] |
| dexa−palo | 1.42 | [0.74; 2.75] |
| busp | 2.23 | [0.29; 17.19] |
| dime−onda | 1.90 | [0.48; 7.55] |
| amis | 1.59 | [0.80; 3.19] |
| cp−12−onda | 3.43 | [0.37; 31.96] |
| cp−12 | 5.82 | [0.70; 48.25] |

Outcome: Headache (including abstracts)

**Comparison: other vs 'plac' (Random Effects Model)**

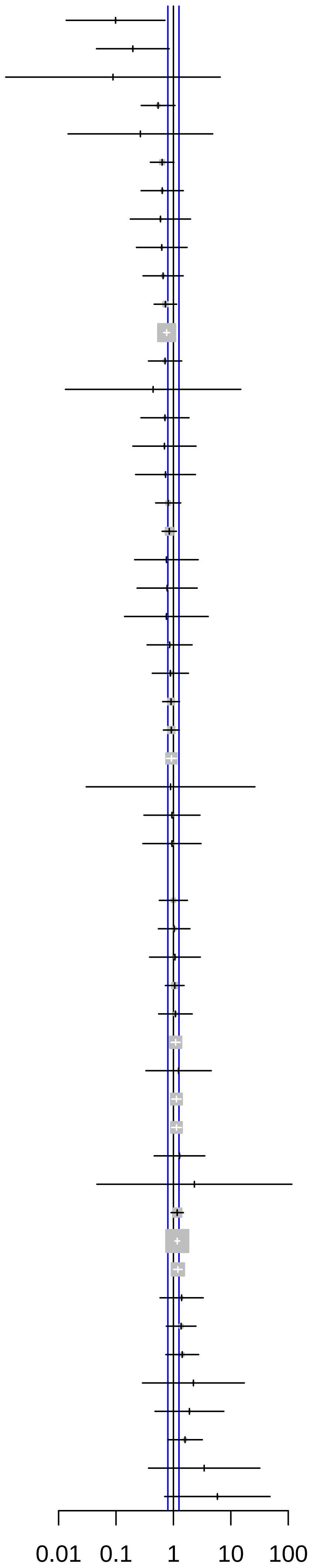


| Treatment | RR | 95%−CI |
|---|---|---|
| sulp | 0.10 | [0.01; 0.71] |
| drop−trop | 0.20 | [0.05; 0.84] |
| dexa−prom | 0.09 | [0.00; 6.54] |
| halo | 0.54 | [0.27; 1.07] |
| prom | 0.27 | [0.01; 4.83] |
| dola−drop | 0.64 | [0.40; 1.02] |
| pred | 0.64 | [0.27; 1.49] |
| drop−onda | 0.59 | [0.18; 1.99] |
| meto−trop | 0.63 | [0.23; 1.73] |
| onda−scop | 0.66 | [0.30; 1.48] |
| dexa−gran | 0.73 | [0.46; 1.14] |
| drop | 0.76 | [0.67; 0.86] |
| halo−onda | 0.72 | [0.37; 1.40] |
| onda−prom | 0.44 | [0.01; 14.90] |
| drop−gran | 0.71 | [0.27; 1.87] |
| dexa−meto−onda | 0.70 | [0.20; 2.48] |
| pene | 0.73 | [0.22; 2.42] |
| caso | 0.81 | [0.49; 1.34] |
| dexa | 0.85 | [0.64; 1.13] |
| dexa−scop | 0.75 | [0.21; 2.71] |
| azas | 0.78 | [0.23; 2.59] |
| apre−palo | 0.75 | [0.14; 4.04] |
| apre | 0.86 | [0.35; 2.11] |
| dexa−drop | 0.88 | [0.43; 1.83] |
| caso−onda | 0.91 | [0.65; 1.28] |
| dexa−onda | 0.92 | [0.67; 1.26] |
| meto | 0.92 | [0.75; 1.13] |
| dexa−halo | 0.89 | [0.03; 26.28] |
| apre−ramo | 0.94 | [0.31; 2.90] |
| meto−onda | 0.94 | [0.29; 3.03] |
| plac | 1.00 | |
| dexa−ramo | 1.00 | [0.57; 1.76] |
| scop | 1.03 | [0.55; 1.94] |
| onda−vest | 1.06 | [0.38; 2.95] |
| apre−dexa | 1.05 | [0.72; 1.53] |
| dexa−meto | 1.08 | [0.55; 2.12] |
| trop | 1.09 | [0.91; 1.31] |
| dime | 1.23 | [0.33; 4.56] |
| dola | 1.13 | [0.93; 1.36] |
| ramo | 1.13 | [0.93; 1.38] |
| pene−trop | 1.27 | [0.46; 3.52] |
| drop−palo | 2.32 | [0.05; 115.63] |
| palo | 1.16 | [0.91; 1.49] |
| onda | 1.17 | [1.06; 1.28] |
| gran | 1.20 | [1.01; 1.42] |
| dixy | 1.39 | [0.58; 3.31] |
| dexa−trop | 1.36 | [0.75; 2.47] |
| dexa−palo | 1.42 | [0.74; 2.75] |
| busp | 2.23 | [0.29; 17.19] |
| dime−onda | 1.90 | [0.48; 7.55] |
| amis | 1.59 | [0.80; 3.19] |
| cp−12−onda | 3.43 | [0.37; 32.00] |
| cp−12 | 5.83 | [0.70; 48.30] |

Outcome: QT prolongation (without abstracts)

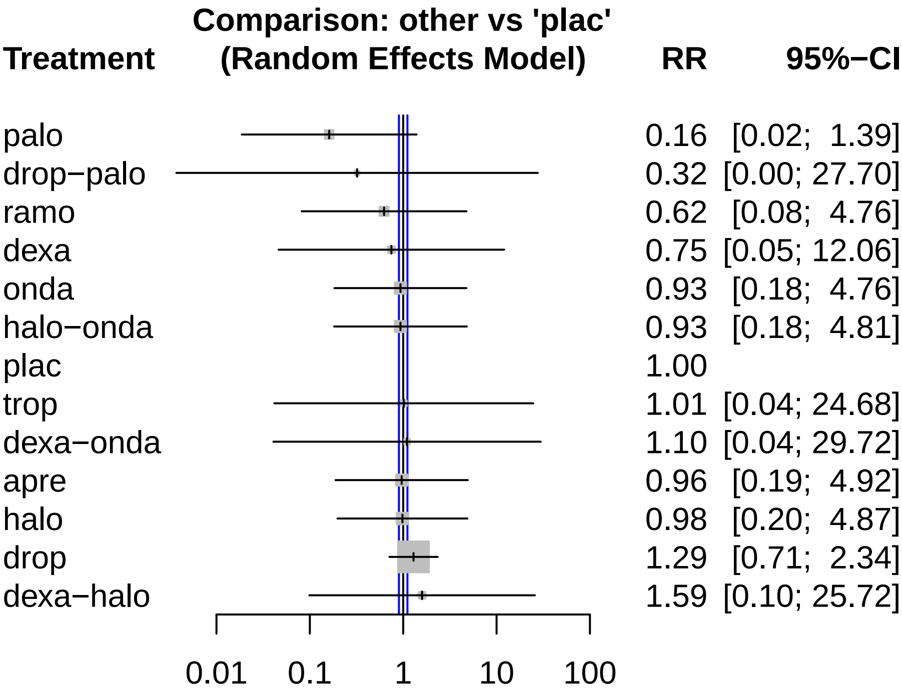

Outcome: QT prolongation (including abstracts)

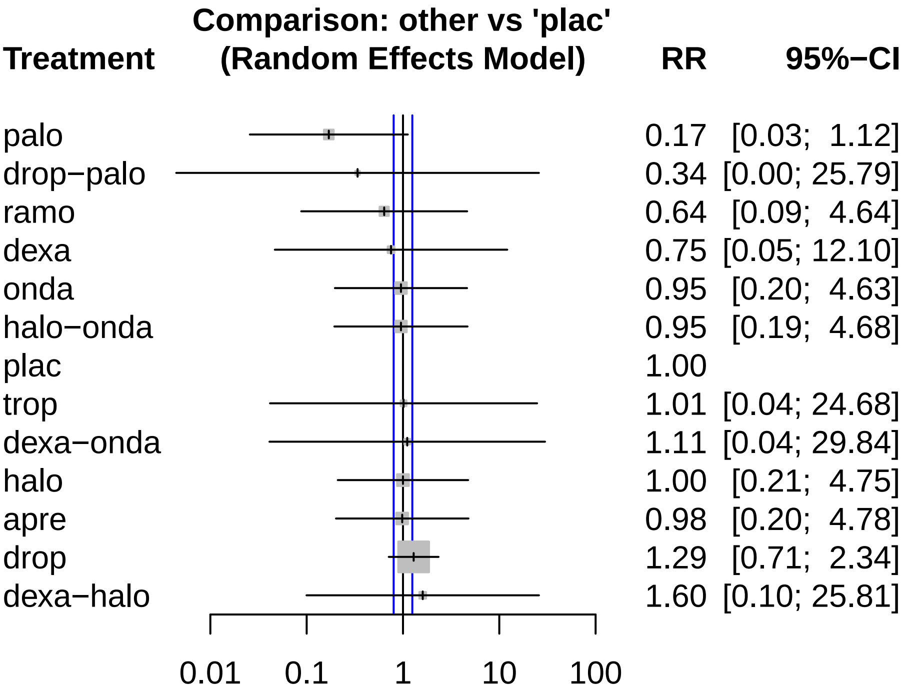

Outcome: Vomiting "early" (without abstracts)

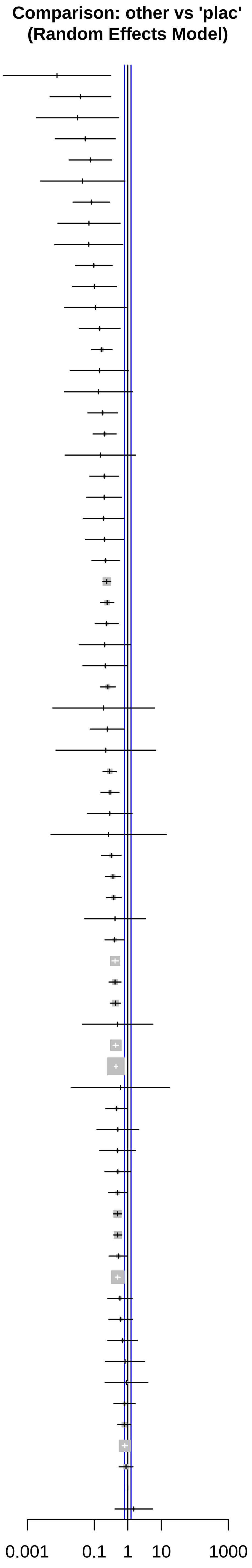


| Treatment | RR | 95%−CI |
|---|---|---|
| apre−palo | 0.01 | [0.00; 0.31] |
| apre−ramo | 0.04 | [0.00; 0.31] |
| dexa−drop−meto−onda | 0.03 | [0.00; 0.54] |
| cp−12−onda | 0.05 | [0.01; 0.43] |
| cycl−onda | 0.08 | [0.02; 0.33] |
| pene−trop | 0.04 | [0.00; 0.83] |
| apre−dexa | 0.08 | [0.02; 0.29] |
| dexa−scop | 0.07 | [0.01; 0.59] |
| apre−onda | 0.07 | [0.01; 0.71] |
| fosa | 0.10 | [0.03; 0.34] |
| benz | 0.10 | [0.02; 0.46] |
| dexa−halo−onda | 0.11 | [0.01; 0.90] |
| apre−dexa−onda | 0.14 | [0.04; 0.59] |
| dexa−drop−onda | 0.17 | [0.08; 0.34] |
| dola−drop | 0.14 | [0.02; 1.06] |
| dexa−ramo | 0.13 | [0.01; 1.37] |
| dexa−dola | 0.18 | [0.06; 0.50] |
| prom | 0.20 | [0.09; 0.46] |
| perp | 0.15 | [0.01; 1.72] |
| dexa−trop | 0.20 | [0.07; 0.54] |
| sulp | 0.20 | [0.06; 0.66] |
| dime−onda | 0.19 | [0.05; 0.78] |
| drop−gran | 0.20 | [0.05; 0.74] |
| cp−12 | 0.22 | [0.09; 0.56] |
| dexa−onda | 0.24 | [0.18; 0.31] |
| dexa−gran | 0.24 | [0.15; 0.38] |
| halo−onda | 0.24 | [0.11; 0.52] |
| apre | 0.21 | [0.04; 1.19] |
| ceti | 0.21 | [0.05; 0.99] |
| drop−onda | 0.26 | [0.15; 0.43] |
| dexa−meto−onda | 0.19 | [0.01; 6.37] |
| drop−meto | 0.24 | [0.08; 0.80] |
| dexa−dola−drop | 0.22 | [0.01; 6.82] |
| dexa−drop | 0.29 | [0.18; 0.47] |
| dexa−palo | 0.29 | [0.16; 0.55] |
| meth | 0.29 | [0.06; 1.34] |
| drop−trop | 0.27 | [0.01; 14.02] |
| dexa−halo | 0.32 | [0.17; 0.63] |
| onda−scop | 0.36 | [0.22; 0.61] |
| dexa−meto | 0.38 | [0.23; 0.64] |
| diph | 0.42 | [0.05; 3.39] |
| halo | 0.41 | [0.21; 0.79] |
| gran | 0.42 | [0.33; 0.52] |
| dola | 0.42 | [0.27; 0.63] |
| ramo | 0.42 | [0.30; 0.61] |
| chlo | 0.50 | [0.04; 5.62] |
| drop | 0.44 | [0.36; 0.53] |
| onda | 0.45 | [0.39; 0.50] |
| meto−onda | 0.60 | [0.02; 17.84] |
| pred | 0.46 | [0.22; 0.97] |
| dixy | 0.51 | [0.12; 2.12] |
| pene | 0.49 | [0.14; 1.69] |
| olan | 0.51 | [0.21; 1.23] |
| cycl | 0.49 | [0.26; 0.92] |
| trop | 0.49 | [0.37; 0.66] |
| palo | 0.50 | [0.38; 0.67] |
| proc | 0.52 | [0.28; 0.99] |
| dexa | 0.50 | [0.43; 0.59] |
| thie | 0.58 | [0.25; 1.36] |
| dime | 0.61 | [0.27; 1.39] |
| meto−scop | 0.71 | [0.25; 1.97] |
| doxy | 0.83 | [0.21; 3.19] |
| beta | 0.91 | [0.21; 3.98] |
| scop | 0.80 | [0.39; 1.66] |
| domp | 0.79 | [0.50; 1.24] |
| meto | 0.82 | [0.68; 0.98] |
| aliz | 0.89 | [0.55; 1.44] |
| plac | 1.00 | |
| perp−scop | 1.50 | [0.41; 5.46] |

Outcome: Vomiting "early" (including abstracts)

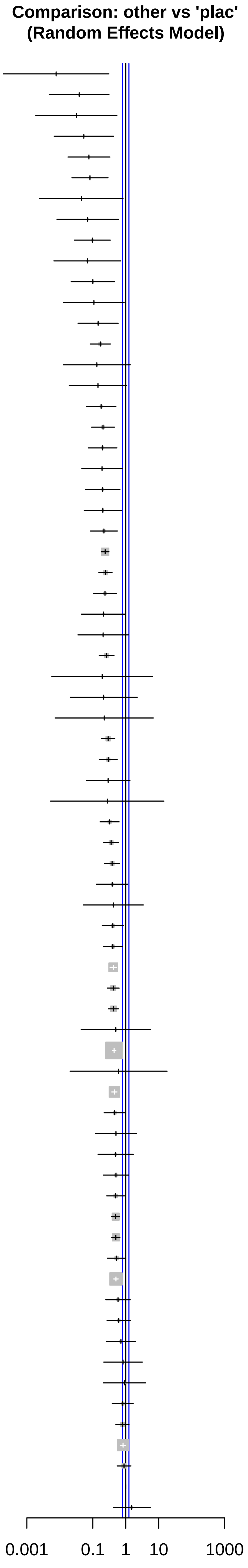


| Treatment | RR | 95%−CI |
|---|---|---|
| apre−palo | 0.01 | [0.00; 0.31] |
| apre−ramo | 0.04 | [0.00; 0.31] |
| dexa−drop−meto−onda | 0.03 | [0.00; 0.54] |
| cp−12−onda | 0.05 | [0.01; 0.43] |
| cycl−onda | 0.08 | [0.02; 0.33] |
| apre−dexa | 0.08 | [0.02; 0.29] |
| pene−trop | 0.04 | [0.00; 0.83] |
| dexa−scop | 0.07 | [0.01; 0.60] |
| fosa | 0.10 | [0.03; 0.34] |
| apre−onda | 0.07 | [0.01; 0.71] |
| benz | 0.10 | [0.02; 0.46] |
| dexa−halo−onda | 0.11 | [0.01; 0.90] |
| apre−dexa−onda | 0.15 | [0.04; 0.59] |
| dexa−drop−onda | 0.17 | [0.08; 0.34] |
| dexa−ramo | 0.13 | [0.01; 1.37] |
| dola−drop | 0.14 | [0.02; 1.07] |
| dexa−dola | 0.18 | [0.06; 0.50] |
| prom | 0.20 | [0.09; 0.46] |
| dexa−trop | 0.20 | [0.07; 0.54] |
| dime−onda | 0.19 | [0.05; 0.78] |
| sulp | 0.20 | [0.06; 0.67] |
| drop−gran | 0.20 | [0.05; 0.75] |
| cp−12 | 0.22 | [0.08; 0.56] |
| dexa−onda | 0.24 | [0.18; 0.31] |
| dexa−gran | 0.24 | [0.15; 0.38] |
| halo−onda | 0.23 | [0.11; 0.52] |
| ceti | 0.21 | [0.05; 0.99] |
| apre | 0.21 | [0.04; 1.19] |
| drop−onda | 0.26 | [0.16; 0.44] |
| dexa−meto−onda | 0.19 | [0.01; 6.42] |
| perp | 0.21 | [0.02; 2.24] |
| dexa−dola−drop | 0.22 | [0.01; 6.86] |
| dexa−drop | 0.29 | [0.18; 0.47] |
| dexa−palo | 0.29 | [0.16; 0.55] |
| meth | 0.29 | [0.06; 1.34] |
| drop−trop | 0.27 | [0.01; 14.40] |
| dexa−halo | 0.32 | [0.17; 0.63] |
| onda−scop | 0.36 | [0.21; 0.61] |
| dexa−meto | 0.38 | [0.23; 0.65] |
| dola−scop | 0.39 | [0.13; 1.15] |
| diph | 0.42 | [0.05; 3.42] |
| drop−meto | 0.41 | [0.19; 0.85] |
| halo | 0.41 | [0.21; 0.79] |
| gran | 0.42 | [0.33; 0.53] |
| dola | 0.42 | [0.27; 0.63] |
| ramo | 0.42 | [0.30; 0.61] |
| chlo | 0.50 | [0.04; 5.63] |
| onda | 0.44 | [0.39; 0.50] |
| meto−onda | 0.61 | [0.02; 17.95] |
| drop | 0.45 | [0.37; 0.55] |
| pred | 0.46 | [0.22; 0.97] |
| dixy | 0.51 | [0.12; 2.13] |
| pene | 0.49 | [0.14; 1.69] |
| olan | 0.51 | [0.21; 1.23] |
| cycl | 0.49 | [0.26; 0.92] |
| trop | 0.49 | [0.37; 0.66] |
| palo | 0.50 | [0.38; 0.67] |
| proc | 0.53 | [0.28; 1.00] |
| dexa | 0.50 | [0.43; 0.59] |
| thie | 0.58 | [0.25; 1.37] |
| dime | 0.61 | [0.27; 1.39] |
| meto−scop | 0.71 | [0.26; 1.99] |
| doxy | 0.83 | [0.21; 3.20] |
| beta | 0.91 | [0.21; 3.99] |
| scop | 0.81 | [0.39; 1.68] |
| domp | 0.79 | [0.50; 1.26] |
| meto | 0.83 | [0.69; 0.99] |
| aliz | 0.89 | [0.55; 1.44] |
| plac | 1.00 | |
| perp−scop | 1.52 | [0.42; 5.53] |

Outcome: Vomiting "late" (without abstracts)

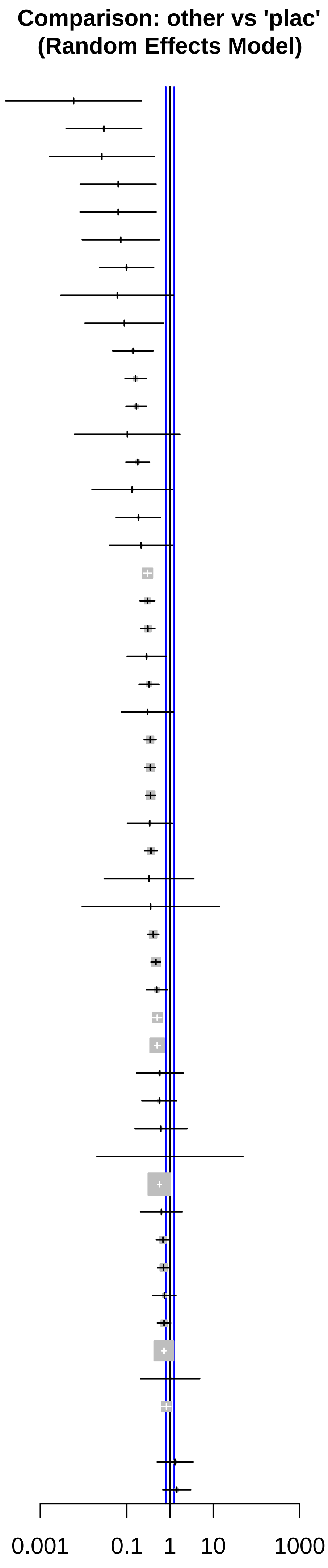


| Treatment | RR | 95%−CI |
|---|---|---|
| apre−palo | 0.01 | [0.00; 0.22] |
| apre−ramo | 0.03 | [0.00; 0.22] |
| dexa−drop−meto−onda | 0.03 | [0.00; 0.43] |
| apre−dexa | 0.06 | [0.01; 0.48] |
| azas | 0.06 | [0.01; 0.48] |
| apre | 0.07 | [0.01; 0.57] |
| cycl−onda | 0.10 | [0.02; 0.42] |
| dexa−dime | 0.06 | [0.00; 1.21] |
| dexa−ramo | 0.09 | [0.01; 0.72] |
| apre−dexa−onda | 0.14 | [0.05; 0.41] |
| dexa−palo | 0.16 | [0.09; 0.28] |
| dexa−gran | 0.17 | [0.10; 0.29] |
| dime−onda | 0.10 | [0.01; 1.71] |
| dexa−dola | 0.18 | [0.10; 0.34] |
| cycl | 0.13 | [0.02; 1.13] |
| halo−onda | 0.19 | [0.06; 0.61] |
| prom | 0.22 | [0.04; 1.16] |
| dexa−onda | 0.30 | [0.24; 0.38] |
| dexa−halo | 0.30 | [0.20; 0.44] |
| dexa−drop−onda | 0.31 | [0.21; 0.45] |
| dexa−halo−onda | 0.29 | [0.10; 0.82] |
| dexa−meto | 0.33 | [0.19; 0.56] |
| pene−trop | 0.30 | [0.08; 1.21] |
| dexa−drop | 0.35 | [0.25; 0.48] |
| gran | 0.35 | [0.26; 0.47] |
| ramo | 0.35 | [0.27; 0.46] |
| meth | 0.34 | [0.10; 1.13] |
| palo | 0.36 | [0.26; 0.52] |
| dexa−dola−drop | 0.33 | [0.03; 3.56] |
| apre−scop | 0.36 | [0.01; 13.75] |
| drop−onda | 0.41 | [0.30; 0.55] |
| onda−scop | 0.47 | [0.36; 0.61] |
| olan | 0.50 | [0.28; 0.88] |
| trop | 0.51 | [0.40; 0.64] |
| dexa | 0.51 | [0.43; 0.59] |
| drop−gran | 0.58 | [0.17; 2.02] |
| drop−meto | 0.57 | [0.22; 1.43] |
| perp | 0.62 | [0.15; 2.48] |
| beta | 1.00 | [0.02; 48.80] |
| onda | 0.57 | [0.51; 0.63] |
| dime | 0.63 | [0.21; 1.93] |
| dola | 0.69 | [0.48; 0.99] |
| halo | 0.71 | [0.52; 0.99] |
| ceti | 0.74 | [0.40; 1.37] |
| scop | 0.73 | [0.51; 1.05] |
| drop | 0.73 | [0.65; 0.81] |
| dixy | 1.01 | [0.21; 4.88] |
| meto | 0.83 | [0.66; 1.04] |
| plac | 1.00 | |
| pene | 1.32 | [0.50; 3.45] |
| pred | 1.44 | [0.68; 3.03] |

Outcome: Vomiting "late" (including abstracts)

Comparison: other vs 'plac'
(Random Effects Model)

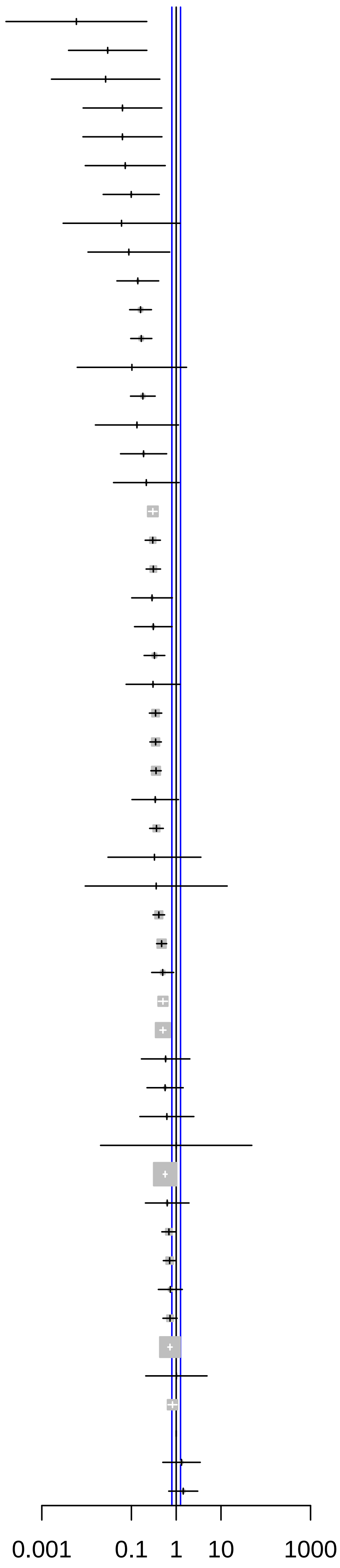


| Treatment | RR | 95%−CI |
|---|---|---|
| apre−palo | 0.01 | [0.00; 0.22] |
| apre−ramo | 0.03 | [0.00; 0.22] |
| dexa−drop−meto−onda | 0.03 | [0.00; 0.43] |
| apre−dexa | 0.06 | [0.01; 0.48] |
| azas | 0.06 | [0.01; 0.48] |
| apre | 0.07 | [0.01; 0.57] |
| cycl−onda | 0.10 | [0.02; 0.42] |
| dexa−dime | 0.06 | [0.00; 1.21] |
| dexa−ramo | 0.09 | [0.01; 0.72] |
| apre−dexa−onda | 0.14 | [0.05; 0.41] |
| dexa−palo | 0.16 | [0.09; 0.28] |
| dexa−gran | 0.17 | [0.10; 0.29] |
| dime−onda | 0.10 | [0.01; 1.71] |
| dexa−dola | 0.18 | [0.10; 0.34] |
| cycl | 0.13 | [0.02; 1.13] |
| halo−onda | 0.19 | [0.06; 0.61] |
| prom | 0.22 | [0.04; 1.16] |
| dexa−onda | 0.30 | [0.24; 0.38] |
| dexa−halo | 0.30 | [0.20; 0.44] |
| dexa−drop−onda | 0.31 | [0.21; 0.45] |
| dexa−halo−onda | 0.29 | [0.10; 0.82] |
| dola−scop | 0.31 | [0.12; 0.81] |
| dexa−meto | 0.33 | [0.19; 0.56] |
| pene−trop | 0.30 | [0.08; 1.21] |
| dexa−drop | 0.35 | [0.25; 0.48] |
| gran | 0.35 | [0.26; 0.47] |
| ramo | 0.35 | [0.27; 0.46] |
| meth | 0.34 | [0.10; 1.13] |
| palo | 0.36 | [0.26; 0.52] |
| dexa−dola−drop | 0.33 | [0.03; 3.56] |
| apre−scop | 0.36 | [0.01; 13.75] |
| drop−onda | 0.41 | [0.30; 0.55] |
| onda−scop | 0.47 | [0.36; 0.61] |
| olan | 0.50 | [0.28; 0.88] |
| trop | 0.51 | [0.40; 0.64] |
| dexa | 0.51 | [0.43; 0.59] |
| drop−gran | 0.58 | [0.17; 2.02] |
| drop−meto | 0.57 | [0.22; 1.43] |
| perp | 0.62 | [0.15; 2.48] |
| beta | 1.00 | [0.02; 48.80] |
| onda | 0.57 | [0.51; 0.63] |
| dime | 0.63 | [0.21; 1.93] |
| dola | 0.69 | [0.48; 0.99] |
| halo | 0.71 | [0.52; 0.99] |
| ceti | 0.74 | [0.40; 1.37] |
| scop | 0.73 | [0.51; 1.05] |
| drop | 0.73 | [0.65; 0.81] |
| dixy | 1.01 | [0.21; 4.88] |
| meto | 0.83 | [0.66; 1.04] |
| plac | 1.00 | |
| pene | 1.32 | [0.50; 3.45] |
| pred | 1.44 | [0.68; 3.03] |

Outcome: Nausea (without abstracts)

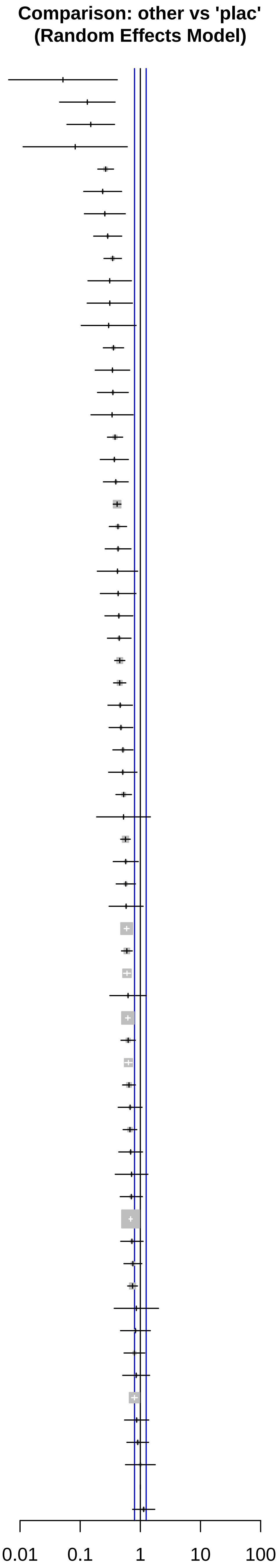


| Treatment | RR | 95%−CI |
|---|---|---|
| dexa−meto−onda | 0.05 | [0.01; 0.41] |
| mecl−onda | 0.13 | [0.05; 0.38] |
| apre−palo | 0.15 | [0.06; 0.37] |
| meto−onda | 0.08 | [0.01; 0.61] |
| dexa−gran | 0.27 | [0.20; 0.36] |
| drop−gran | 0.24 | [0.11; 0.49] |
| apre−dexa−onda | 0.26 | [0.12; 0.56] |
| dexa−dime | 0.29 | [0.17; 0.49] |
| dexa−halo | 0.35 | [0.25; 0.49] |
| meto−trop | 0.31 | [0.13; 0.71] |
| dixy | 0.31 | [0.13; 0.74] |
| dexa−dola−drop | 0.30 | [0.10; 0.85] |
| dexa−palo | 0.36 | [0.24; 0.53] |
| dexa−drop−meto−onda | 0.34 | [0.18; 0.67] |
| apre−ramo | 0.35 | [0.19; 0.63] |
| onda−prom | 0.34 | [0.15; 0.76] |
| dexa−drop−onda | 0.38 | [0.28; 0.51] |
| dola−drop | 0.37 | [0.22; 0.63] |
| dexa−scop | 0.39 | [0.24; 0.63] |
| dexa−onda | 0.41 | [0.35; 0.48] |
| dexa−trop | 0.42 | [0.30; 0.59] |
| dexa−ramo | 0.43 | [0.26; 0.70] |
| dexa−prom | 0.42 | [0.19; 0.90] |
| halo−onda | 0.43 | [0.22; 0.85] |
| dexa−dola | 0.44 | [0.26; 0.75] |
| meth | 0.45 | [0.28; 0.70] |
| drop−onda | 0.46 | [0.37; 0.55] |
| dexa−drop | 0.45 | [0.36; 0.57] |
| halo−trop | 0.46 | [0.29; 0.74] |
| apre−dexa | 0.48 | [0.30; 0.75] |
| onda−scop | 0.51 | [0.35; 0.76] |
| dime | 0.51 | [0.30; 0.88] |
| dexa−meto | 0.53 | [0.39; 0.71] |
| meth−trop | 0.53 | [0.19; 1.47] |
| ramo | 0.57 | [0.47; 0.68] |
| apre | 0.57 | [0.35; 0.93] |
| prom | 0.57 | [0.40; 0.83] |
| proc | 0.58 | [0.30; 1.11] |
| drop | 0.59 | [0.54; 0.65] |
| palo | 0.60 | [0.49; 0.73] |
| gran | 0.60 | [0.52; 0.69] |
| drop−trop | 0.63 | [0.31; 1.26] |
| dexa | 0.62 | [0.57; 0.68] |
| scop | 0.63 | [0.48; 0.83] |
| trop | 0.63 | [0.55; 0.73] |
| caso−onda | 0.65 | [0.51; 0.83] |
| cp−12 | 0.68 | [0.43; 1.07] |
| halo | 0.67 | [0.52; 0.88] |
| cp−12−onda | 0.69 | [0.44; 1.09] |
| drop−meto | 0.72 | [0.38; 1.34] |
| fosa | 0.71 | [0.46; 1.08] |
| onda | 0.69 | [0.65; 0.74] |
| caso | 0.72 | [0.47; 1.11] |
| amis | 0.75 | [0.53; 1.06] |
| dola | 0.74 | [0.62; 0.90] |
| trbe | 0.86 | [0.37; 2.01] |
| beta | 0.83 | [0.47; 1.47] |
| rola | 0.80 | [0.54; 1.19] |
| pred | 0.85 | [0.51; 1.43] |
| meto | 0.80 | [0.72; 0.89] |
| aliz | 0.87 | [0.55; 1.38] |
| domp | 0.91 | [0.60; 1.37] |
| busp | 1.00 | [0.57; 1.77] |
| plac | 1.00 | |
| cycl | 1.14 | [0.74; 1.74] |

Outcome: Nausea (including abstracts)

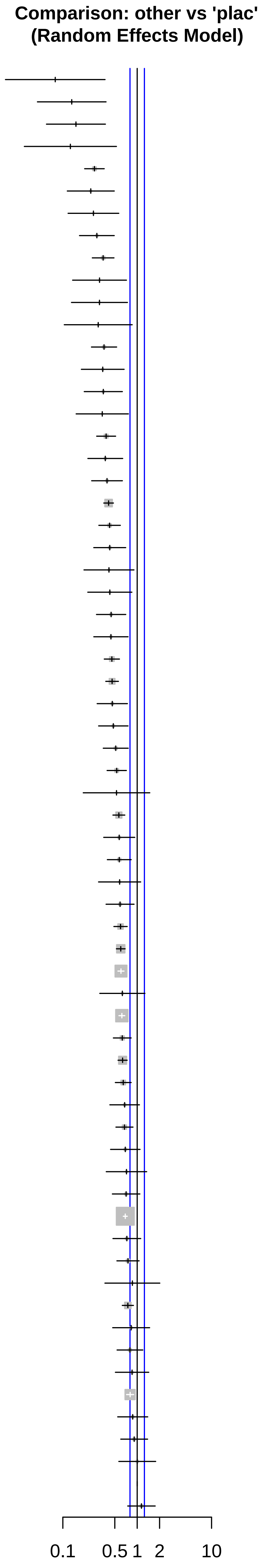


| Treatment | RR | 95%−CI |
|---|---|---|
| dexa−meto−onda | 0.08 | [0.02; 0.37] |
| mecl−onda | 0.13 | [0.05; 0.38] |
| apre−palo | 0.15 | [0.06; 0.37] |
| meto−onda | 0.13 | [0.03; 0.53] |
| dexa−gran | 0.27 | [0.20; 0.36] |
| drop−gran | 0.24 | [0.11; 0.49] |
| apre−dexa−onda | 0.26 | [0.12; 0.57] |
| dexa−dime | 0.29 | [0.17; 0.49] |
| dexa−halo | 0.35 | [0.25; 0.49] |
| meto−trop | 0.31 | [0.14; 0.72] |
| dixy | 0.31 | [0.13; 0.74] |
| dexa−dola−drop | 0.30 | [0.10; 0.86] |
| dexa−palo | 0.36 | [0.24; 0.53] |
| dexa−drop−meto−onda | 0.34 | [0.18; 0.67] |
| apre−ramo | 0.35 | [0.19; 0.63] |
| onda−prom | 0.34 | [0.15; 0.76] |
| dexa−drop−onda | 0.38 | [0.29; 0.51] |
| dola−drop | 0.37 | [0.22; 0.64] |
| dexa−scop | 0.39 | [0.24; 0.63] |
| dexa−onda | 0.41 | [0.36; 0.48] |
| dexa−trop | 0.43 | [0.30; 0.59] |
| dexa−ramo | 0.43 | [0.26; 0.70] |
| dexa−prom | 0.42 | [0.19; 0.90] |
| halo−onda | 0.43 | [0.22; 0.85] |
| meth | 0.45 | [0.28; 0.70] |
| dexa−dola | 0.44 | [0.26; 0.75] |
| dexa−drop | 0.46 | [0.36; 0.58] |
| drop−onda | 0.46 | [0.38; 0.56] |
| halo−trop | 0.46 | [0.29; 0.74] |
| apre−dexa | 0.48 | [0.30; 0.75] |
| onda−scop | 0.51 | [0.35; 0.76] |
| dexa−meto | 0.53 | [0.39; 0.71] |
| meth−trop | 0.53 | [0.19; 1.48] |
| ramo | 0.57 | [0.47; 0.68] |
| apre | 0.57 | [0.35; 0.93] |
| prom | 0.57 | [0.40; 0.83] |
| proc | 0.58 | [0.30; 1.11] |
| dime | 0.59 | [0.38; 0.91] |
| palo | 0.60 | [0.49; 0.73] |
| gran | 0.60 | [0.53; 0.69] |
| drop | 0.60 | [0.55; 0.67] |
| drop−trop | 0.63 | [0.31; 1.27] |
| dexa | 0.62 | [0.57; 0.68] |
| scop | 0.63 | [0.48; 0.83] |
| trop | 0.64 | [0.55; 0.73] |
| caso−onda | 0.65 | [0.51; 0.83] |
| cp−12 | 0.68 | [0.43; 1.07] |
| halo | 0.67 | [0.52; 0.88] |
| cp−12−onda | 0.69 | [0.44; 1.09] |
| drop−meto | 0.72 | [0.38; 1.34] |
| fosa | 0.71 | [0.46; 1.09] |
| onda | 0.69 | [0.65; 0.74] |
| caso | 0.73 | [0.47; 1.12] |
| amis | 0.75 | [0.53; 1.06] |
| trbe | 0.86 | [0.37; 2.01] |
| dola | 0.75 | [0.63; 0.89] |
| beta | 0.83 | [0.47; 1.47] |
| rola | 0.80 | [0.54; 1.19] |
| pred | 0.85 | [0.51; 1.43] |
| meto | 0.80 | [0.72; 0.90] |
| aliz | 0.87 | [0.55; 1.39] |
| domp | 0.91 | [0.60; 1.38] |
| busp | 1.00 | [0.57; 1.77] |
| plac | 1.00 | |
| cycl | 1.14 | [0.75; 1.75] |

Outcome: Complete response (without abstracts)

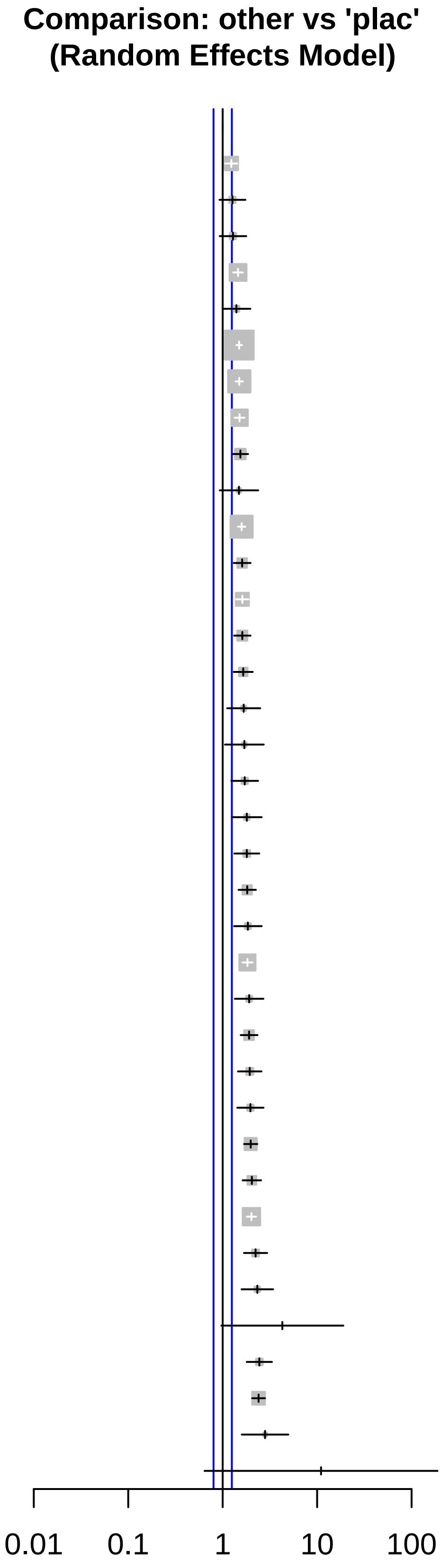


| Treatment | RR | 95%−CI |
|---|---|---|
| plac | 1.00 | |
| meto | 1.23 | [1.06; 1.43] |
| dixy | 1.27 | [0.92; 1.74] |
| amis | 1.29 | [0.93; 1.78] |
| dola | 1.45 | [1.29; 1.64] |
| rola | 1.40 | [0.99; 1.96] |
| onda | 1.49 | [1.40; 1.60] |
| drop | 1.50 | [1.37; 1.64] |
| trop | 1.51 | [1.34; 1.70] |
| halo | 1.54 | [1.28; 1.86] |
| apre−scop | 1.49 | [0.93; 2.37] |
| dexa | 1.59 | [1.45; 1.74] |
| dexa−meto | 1.61 | [1.31; 1.97] |
| palo | 1.62 | [1.39; 1.88] |
| dola−drop | 1.61 | [1.32; 1.97] |
| apre | 1.65 | [1.31; 2.08] |
| apre−dexa | 1.67 | [1.12; 2.50] |
| dexa−dola | 1.70 | [1.06; 2.72] |
| caso | 1.71 | [1.24; 2.37] |
| dexa−drop | 1.80 | [1.25; 2.59] |
| fosa | 1.80 | [1.33; 2.44] |
| drop−onda | 1.82 | [1.47; 2.25] |
| onda−scop | 1.85 | [1.32; 2.59] |
| gran | 1.83 | [1.62; 2.07] |
| onda−vest | 1.91 | [1.35; 2.70] |
| dexa−palo | 1.90 | [1.55; 2.33] |
| dexa−trop | 1.93 | [1.45; 2.58] |
| halo−onda | 1.97 | [1.43; 2.71] |
| caso−onda | 1.98 | [1.68; 2.33] |
| dexa−halo | 2.04 | [1.63; 2.55] |
| dexa−onda | 2.02 | [1.80; 2.26] |
| ramo | 2.23 | [1.68; 2.96] |
| drop−palo | 2.33 | [1.58; 3.42] |
| dexa−drop−onda | 4.29 | [0.97; 18.96] |
| drop−gran | 2.45 | [1.80; 3.33] |
| dexa−gran | 2.40 | [2.05; 2.80] |
| cycl−onda | 2.81 | [1.59; 4.96] |
| scop | 11.00 | [0.64; 188.18] |

Outcome: Complete response (including abstracts)

**Comparison: other vs 'plac' (Random Effects Model)**

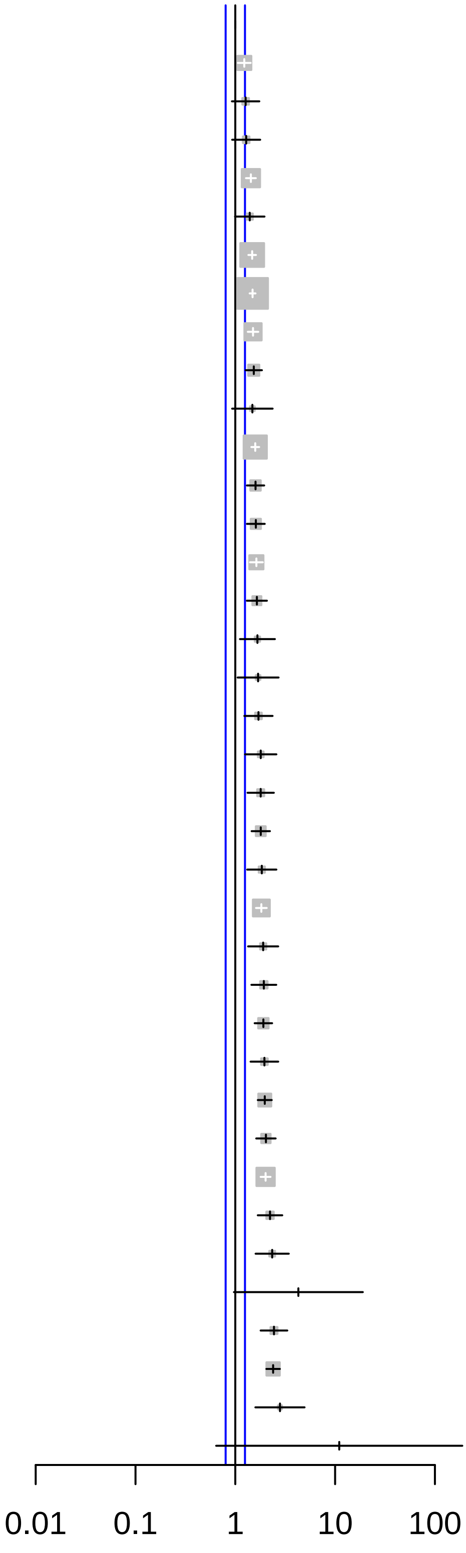


| Treatment | RR | 95%−CI |
|---|---|---|
| plac | 1.00 | |
| meto | 1.23 | [1.06; 1.43] |
| dixy | 1.27 | [0.92; 1.74] |
| amis | 1.29 | [0.93; 1.78] |
| dola | 1.43 | [1.28; 1.61] |
| rola | 1.40 | [0.99; 1.96] |
| drop | 1.48 | [1.35; 1.61] |
| onda | 1.49 | [1.39; 1.59] |
| trop | 1.51 | [1.33; 1.70] |
| halo | 1.53 | [1.27; 1.85] |
| apre−scop | 1.48 | [0.93; 2.37] |
| dexa | 1.58 | [1.45; 1.73] |
| dola−drop | 1.59 | [1.31; 1.95] |
| dexa−meto | 1.61 | [1.31; 1.97] |
| palo | 1.63 | [1.40; 1.89] |
| apre | 1.65 | [1.31; 2.07] |
| apre−dexa | 1.67 | [1.11; 2.49] |
| dexa−dola | 1.69 | [1.05; 2.71] |
| caso | 1.71 | [1.23; 2.36] |
| dexa−drop | 1.80 | [1.25; 2.58] |
| fosa | 1.80 | [1.33; 2.43] |
| drop−onda | 1.80 | [1.46; 2.22] |
| onda−scop | 1.84 | [1.32; 2.58] |
| gran | 1.82 | [1.61; 2.06] |
| onda−vest | 1.90 | [1.34; 2.69] |
| dexa−trop | 1.93 | [1.45; 2.57] |
| dexa−palo | 1.91 | [1.56; 2.34] |
| halo−onda | 1.96 | [1.42; 2.69] |
| caso−onda | 1.97 | [1.68; 2.32] |
| dexa−halo | 2.03 | [1.62; 2.54] |
| dexa−onda | 2.01 | [1.79; 2.25] |
| ramo | 2.23 | [1.68; 2.96] |
| drop−palo | 2.34 | [1.60; 3.43] |
| dexa−drop−onda | 4.29 | [0.97; 18.96] |
| drop−gran | 2.44 | [1.79; 3.32] |
| dexa−gran | 2.39 | [2.05; 2.80] |
| cycl−onda | 2.80 | [1.59; 4.94] |
| scop | 11.00 | [0.64; 188.18] |
